# Soluble mutant huntingtin drives early human pathogenesis in Huntington’s disease

**DOI:** 10.1007/s00018-023-04882-w

**Published:** 2023-08-03

**Authors:** Andrés Miguez, Cinta Gomis, Cristina Vila, Marta Monguió-Tortajada, Sara Fernández-García, Georgina Bombau, Mireia Galofré, María García-Bravo, Phil Sanders, Helena Fernández-Medina, Blanca Poquet, Cristina Salado-Manzano, Santiago Roura, Jordi Alberch, José Carlos Segovia, Nicholas D. Allen, Francesc E. Borràs, Josep M. Canals

**Affiliations:** 1 Laboratory of Stem Cells and Regenerative Medicine, Department of Biomedical Sciences, Faculty of Medicine and Health Sciences, University of Barcelona, Barcelona, Spain; 2 Creatio, Production and Validation Center of Advanced Therapies, Faculty of Medicine and Health Sciences, University of Barcelona, Barcelona, Spain; 3 Institute of Neurosciences, University of Barcelona, Barcelona, Spain; 4 August Pi i Sunyer Biomedical Research Institute (IDIBAPS), Barcelona, Spain; 5 Networked Biomedical Research Centre for Neurodegenerative Disorders (CIBERNED), Madrid, Spain; 6 REMAR-IVECAT Group, Germans Trias i Pujol Health Science Research Institute, Can Ruti Campus, Badalona, Spain; 7 Laboratory of Pathophysiology of Neurodegenerative Diseases, Department of Biomedical Sciences, Faculty of Medicine and Health Sciences, University of Barcelona, Barcelona, Spain; 8 Division of Hematopoietic Innovative Therapies, Centro de Investigaciones Energéticas, Medioambientales y Tecnológicas, Madrid, Spain; 9 ICREC Research Program, Germans Trias i Pujol Health Science Research Institute, Can Ruti Campus, Badalona, Spain; 10 Faculty of Medicine, University of Vic-Central University of Catalonia (UVic-UCC), Vic, Spain; 11 Brain Repair Group, School of Biosciences, Cardiff University, Cardiff, UK; 12 Nephrology Department, Germans Trias i Pujol Universitary Hospital, Badalona, Spain; 13 Present Address: Neurology-Neuroimmunology Department, Multiple Sclerosis Centre of Catalunya (Cemcat), Vall d’Hebron Research Institute (VHIR), Vall d’Hebron University Hospital, Barcelona, Spain

**Keywords:** Disease modelling, Cell transplantation, Oligomers, Extracellular vesicles, Neurodegeneration, Induced pluripotent stem cells

## Abstract

Huntington's disease (HD) is an incurable inherited brain disorder characterised by massive degeneration of striatal neurons, which correlates with abnormal accumulation of misfolded mutant huntingtin (mHTT) protein. Research on HD has been hampered by the inability to study early dysfunction and progressive degeneration of human striatal neurons in vivo. To investigate human pathogenesis in a physiologically relevant context, we transplanted human pluripotent stem cell-derived neural progenitor cells (hNPCs) from control and HD patients into the striatum of new-born mice. Most hNPCs differentiated into striatal neurons that projected to their target areas and established synaptic connexions within the host basal ganglia circuitry. Remarkably, HD human striatal neurons first developed soluble forms of mHTT, which primarily targeted endoplasmic reticulum, mitochondria and nuclear membrane to cause structural alterations. Furthermore, HD human cells secreted extracellular vesicles containing mHTT monomers and oligomers, which were internalised by non-mutated mouse striatal neurons triggering cell death. We conclude that interaction of mHTT soluble forms with key cellular organelles initially drives disease progression in HD patients and their transmission through exosomes contributes to spread the disease in a non-cell autonomous manner.

**Graphical abstract:**

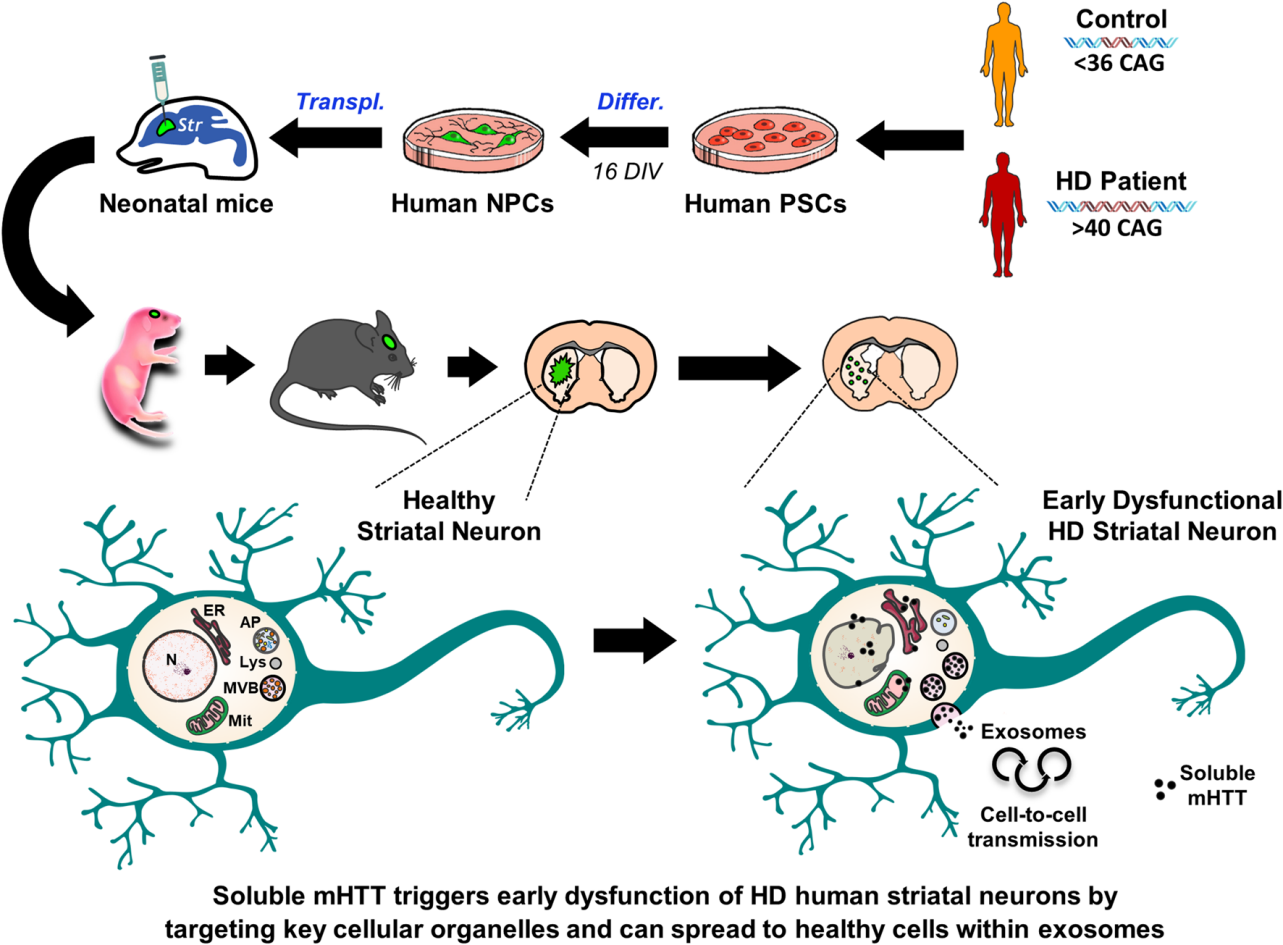

**Supplementary Information:**

The online version contains supplementary material available at 10.1007/s00018-023-04882-w.

## Introduction

Abnormal accumulation of misfolded proteins in the brain is a common feature of many neurodegenerative disorders, including Parkinson’s, Alzheimer’s and Huntington's diseases (HD) [1]. Impaired clearance of these proteins results in the build-up of toxic species, such as oligomers and aggregates, which are associated to neuronal dysfunction and ultimately cell death. Furthermore, emerging evidence in patients and animal models indicates that propagation of misfolded proteins also contributes to the neurodegenerative process [2]. Development of more effective therapies for neurodegenerative diseases requires a better understanding of which are the most toxic protein species, when and where they accumulate, and how they spread throughout the brain. Most importantly, due to the complex pathophysiology of brain disorders, these crucial issues need to be addressed in human cells.

HD is a hereditary neurological disorder caused by a CAG repeat-expansion in the *huntingtin* gene (> 40 CAGs) that mainly provokes striatal atrophy and degeneration of medium spiny neurons (MSNs) [3], resulting in motor and cognitive deficits. There is an inverse correlation between the number of CAG repeats and the age of symptom onset, with larger CAG repeat expansions being associated with earlier ages of onset [4]. HD pathology is linked to the progressive deregulation of multiple cellular processes by mutant huntingtin (mHTT), including proteostasis, autophagy, calcium homeostasis and synaptic plasticity [5]. In addition, recent research suggests that mHTT can be secreted [6] and propagated by means of synaptic transmission [7] or extracellular vesicles (EVs) [8], thereby contributing to non-cell autonomous pathology.

Due to the limited access to patients’ brain tissue, most of our understanding of mechanisms of HD pathogenesis comes from the study of animal models at early disease stages. Although mouse models are valuable tools that mimic several aspects of HD progression, they do not completely match the human condition because of a much higher number of CAG repeats needed to develop the disease, the different pattern of mHTT aggregates and the absence of cell death [9]. Indeed, these differences may account for the low success rates in translating preclinical findings to clinical trials in HD over the last 20 years [10].

HD patient-derived human-induced pluripotent stem cells (hiPSCs) or human embryonic stem cells (hESCs) have been used to examine human pathology in vitro [11–14], as they carry the genetic alterations that contribute to disease. However, in vitro differentiation protocols have the disadvantage of depriving cells of their natural environment, which is critical for neuronal development and ageing. Consequently, most HD hiPSC-based in vitro models are free of mHTT aggregates and lack an overt cell death phenotype, instead showing only subtle neurodegeneration [15]. Alternatively, the in vivo functional integration of HD human pluripotent stem cell (hPSC)-derived neuronal cells within the mouse brain could allow the study of the initiation, progression and full manifestation of HD. To this aim, we transplanted control (CTR-) and HD patient-derived human neural progenitor cells (HD-hNPCs) into the developing mouse striatum and examined long-term differentiation, brain connectivity and neurodegeneration.

Neonatally engrafted HD-hNPCs differentiated into striatal neurons that projected to their target areas and established synaptic connexions within the host basal ganglia circuitry. HD human neurons developed progressive human-specific pathological features, ranging from early intracellular abnormalities to MSN death. Remarkably, we identified soluble mHTT as the primary toxic form on structurally altered endoplasmic reticulum (ER), mitochondria and nuclear membrane. Our study further shows evidence of soluble mHTT spreading through exosomes, as a mechanism of disease propagation.

## Materials and methods

### Mice

*C57BL/6J* pregnant females were obtained from Charles River and *Rag2*^*−/−*^ mice were a kind gift from Dr. Anna Planas (IIBB-CSIC, IDIBAPS, Barcelona, Spain). P2 neonatal mice were used in cell transplantation experiments, whereas E18.5 embryos were employed for primary striatal cell cultures. On weaning, male and female transplanted mice were randomly assigned to matched groups of mixed cell genotypes for either behavioural or histological analysis. Animals were housed with access to food and water ad libitum in a colony room kept at 19–22 °C and 40–60% humidity, under a 12:12 h light/dark cycle. All procedures involving the use of animals were approved by the Animal Experimentation Ethics Committee of the University of Barcelona and Generalitat de Catalunya (10995) in compliance with the Spanish (RD 53/2013 and RD 1386/2018) and European (2010/63/EU) regulations for the care and use of experimental animals.

### In vitro differentiation of human-induced pluripotent stem cell lines

Several hPSC lines were used in the present study under the ethical permission 0336/5796/2019 (Generalitat de Catalunya, Spain). HiPSC lines CTR-33 (CS83iCTR-33n1) and HD-60 (CS21iHD-60n5) (kind gift from C. N. Svendsen, Cedar Sinai, Los Angeles, CA, USA) were generated from human fibroblasts as previously described [12]; hiPSC lines CTR-2190 (hPSCreg: CHDIi042-A) and HD-2174 (hPSCreg; CHDIi026-A) were generously obtained from CHDI Foundation Inc.; and hESCs GEN-019 (hPSCreg; GENEAe020-A) and GEN-020 (hPSCreg; GENEAe015-A) were acquired from Genea Biocells (Australia). All these cell lines passed our standard quality controls defined in Creatio’s quality system (ISO9001:2015). None of the cell lines showed karyotyping alterations except the CTR-2190, that showed a mosaic trisomy in chromosome 12 (75% of the analysed cells) when they were expanded for more than 30 passages in vitro. To track cell migration and neuronal projections after cell implantation, hPSC lines CTR-33 and HD-60 were transduced with a GFP lentivirus under the control of the constitutive EF1-α promoter before in vitro differentiation. Stem cell differentiation towards hNPCs with a ventral forebrain phenotype was performed during 16 DIV, as described elsewhere [16]. At this stage, we evaluated the proliferation of hNPCs from all cell lines by immunocytochemistry against Ki67. The percentage of proliferating cells was calculated by counting the number of Ki67 positive cells out of the total number of cells in the culture evaluated by 4ʹ,6-diamidino-2-fenilindol (DAPI) staining. Stem cell differentiations were conducted under ISO9001-2015 and comply with the Guidance Document on Good In Vitro Method Practices (GIVIMP; OECD) recommendations.

### Cell transplantation

Prior to transplantation, cells were disaggregated with accutase and re-suspended in phosphate-buffered saline (PBS). P2 neonatal mice were anaesthetised by hypothermia, placing them on ice until cessation of movement. Unilateral striatal injections were performed using a stereotaxic apparatus (RWD) coupled to a stereotaxic pump (WPI) and a 10 μl Hamilton syringe with a 33 gauge needle, setting the following coordinates (mm): antero-posterior, + 2.3 from lambda; medio-lateral, + 1.4 from lambda; dorso-ventral, − 1.8 from dura. Every animal received 15,000 cells diluted in 1 μl of PBS and injected at a rate of 0.2 µl/min.

### Mouse behaviour

Amphetamine-induced rotation test was performed to assess circling behaviour as a readout of unilateral striatal degeneration in mice transplanted with HD-60-derived hNPCs, CTR-33-derived hNPCs or sham (injected with PBS). We delivered intraperitoneally 5 mg/kg of amphetamine diluted in 0.9% NaCl. After 2 min of latency, the number of ipsilateral and contralateral turns were counted manually during 15 min. Tracking of mouse behaviour was conducted blinded to experimental condition with the help of SMART software (Panlab).

### Immunohistochemistry

Anaesthetised mice were intra-cardially perfused with PBS and a 4% paraformaldehyde (PFA) solution in 0.1 M phosphate buffer. Brains were cryoprotected with 30% sucrose in PBS, frozen in methylbutane (Sigma) and sectioned. Serial coronal sections (20 μm) of the brain were obtained using a cryostat (Thermo Fisher). Tissue was sequentially incubated with a blocking solution (PBS, 0.3% Triton X-100, 5% Normal horse serum) for 2 h at room temperature and with the primary antibodies (Supplementary Table 3) overnight at 4 °C. After 3 washes with PBS, tissue was incubated for 1 h30ʹ at room temperature with specific fluorescent secondary antibodies. As GFP expression in grafted cells was not ubiquitous, human cells were also identified based on their expression of human nuclear antigen (hNA) and the cytoplasmic marker STEM121. Immunofluorescence images were acquired with a Confocal Leica TCS SP5 microscope and quantified using ImageJ and Computer-Assisted Stereology Toolbox (Olympus Danmark A/S) software. Immuno-labelled cells in the regions of interest were counted on five evenly spaced coronal sections from each mouse. We used high intensity projection of Z stacked images when these were acquired at high magnification (40×, 63×). Stereological estimation of striatal volume was carried out by measuring the striatal area of 10 sections per animal spaced 240 μm apart. GFAP and Iba1 immunoreactivity surrounding the bulk of the graft was quantified by analysis of integrated optical density (ImageJ). Graft size was calculated by delineating the outer perimeter of the STEM121^+^ graft core and estimating its volume through extrapolation of the area quantified in sections spaced 120 μm apart, as previously described [16]. Striatal necrosis was assessed by digitally tracing those striatal regions characterised by tissue disruption and non-specific DARPP-32 aberrant immunoreactivity, measuring their area with ImageJ software. Spreading of cleaved caspase-3 staining from the bulk of the graft was quantified using plot profile analysis (ImageJ).

### Immunogold labelling and transmission electron microscopy

For TEM studies, samples were fixed with a solution of 2% PFA/0.5% glutaraldehyde in 0.1 M PB, post-fixed with 1% osmium tetroxide, dehydrated and embedded in epoxy resin. Ultra-thin sections (70 nm) were immuno-labelled with primary antibody, followed by incubation with a secondary antibody conjugated with electron-dense colloidal gold nanoparticles of 10 nm size (Aurion, Electron Microscopy Sciences). GFP and STEM121 antibodies were used for detecting human cells, MW1 to identify soluble mHTT (monomers and oligomers), 3B5H10 to label mHTT monomers and EM48 to detect aggregated mHTT species. Images were acquired with a JEOL 1010 transmission electron microscope equipped with a CCD Orius camera (Gatan). For TEM immunogold analysis of human cells and mHTT species, we examined a minimum of 6 ultra-thin sections per animal, at both striatal and external globus pallidus (GPe) levels, for identifying 30 positive cell profiles per each transplanted mouse.

### Extracellular vesicle isolation, characterisation and labelling

EVs were isolated from the conditioned medium of CTR- and HD-derived hNPCs by size-exclusion chromatography (SEC), as described previously [17, 18]. The supernatant of cells cultured in serum-free medium was collected at 16 DIV, centrifuged and concentrated by 100 kDa ultrafiltration at 2000*g* for 35 min with Amicon Ultra (Millipore). EVs were then isolated by elution of concentrated conditioned medium in a 1 ml-SEC of Sepharose CL-2B (Sigma) with PBS (Oxoid), collecting 100 µl-fractions. Protein elution was checked by reading absorbance at 280 nm of each SEC fraction using Nanodrop (Thermo Scientific). EV-enriched fractions were determined by bead-based flow cytometry, as described elsewhere [18]. Briefly, SEC fractions were coupled to 4 μm aldehyde/sulphate-latex microspheres (Invitrogen) and labelled with CD63 (Clone TEA3/18) and CD81 (Clone 5A6) antibodies, because EVs are enriched in these tetraspanin proteins. Data was acquired in a FACSVerse flow cytometer (BD) and analysed by FlowJo v.X software (TreeStar). EV fluorescent labelling with carboxyfluorescein succinimidyl ester (CFSE) (Molecular Probes) was performed by incubating EVs with CFSE (20 µM) for 2 h at 37 °C. Unbound dye was removed by four sequential washes with PBS and 100 kD ultrafiltration.

### Co-culture of mouse striatal neurons with human extracellular vesicles

Brains from E18.5 *C57BL/6J* mouse embryos were excised and placed in Neurobasal medium (Gibco). Dissected striata were gently dissociated and seeded onto 12 mm glass coverslips pre-coated with 0.1 mg/ml poly-d-lysine (Sigma). At 14 DIV, striatal primary cultures were treated with EVs in a 2:1 ratio (EV-donor cells: EV-recipient cells). Specifically, human EVs isolated from the conditioned medium of 3 × 10^5^ donor cells (HD- and CTR-NPCs) were co-cultured with 1.5 × 10^5^ recipient cells (mouse primary neurons). 24 h later, cultures were fixed with PFA 4% and processed for immunocytochemistry. Images were acquired with a Confocal Zeiss LSM 880 microscope and assessment of nuclear size and morphology was performed with ImageJ software. Size of pyknotic neuronal nuclei was established in a range from 7 to 20 µm^2^. DAPI positive particles smaller than 7 µm^2^ or with less than 0.4 circularity were considered artefacts and discarded.

### Pharmacological treatment in vivo

Chronic pharmacological treatment with FTY720 was performed as previously described [19]. FTY720 was obtained as a powder (Cayman Chemicals) and dissolved in EtOH 10% in distilled water (vehicle). Both CTR-33 and HD-60 chimeric mice received intraperitoneal injections of either FTY720 or vehicle solution every four days during two months, at a dose of 0.3 mg/kg.

### Statistical analysis

Statistical analyses were performed with GraphPad Prism 6 software. Values are shown as the mean ± standard error of the mean (SEM). Unpaired two-tailed *t* test was used for simple comparisons of one variable between two groups and one-way ANOVA or two-way ANOVA were used to determine differences between more than two groups, unless otherwise indicated in figure legends. The level of statistical significance was set as follows: *P < 0.05, **P < 0.01, ***P < 0.001.

## Results

### Neonatally engrafted HD patient-derived human neural progenitors show increased proliferation and accelerated MSN differentiation

To explore whether neonatally engrafted hNPCs could expand and populate the mouse striatum, we used 6 hPSC lines (4 hiPSC lines and 2 sibling hESCs). 3 of them were considered control cell lines (< 36 CAGs): CTR-33, CTR-2190 and GEN-019; whilst the other cell lines exceeded the pathological repeat length (> 40 CAGs): HD-2174 (47 CAGs), GEN-020 (48 CAGs) and HD-60 (60 CAGs). HD and CTR hPSCs were differentiated for 16 days in vitro (DIV) to hNPCs as previously described [16], before being unilaterally transplanted into the striatum of neonatal wild-type (WT) mice (Supplementary Fig. 1). Implanted cells were unambiguously identified based on their expression of human nuclear antigen (hNA) and the human cytoplasmic marker STEM121. We observed cell engraftment and survival up to 5 months post-transplantation (PST) for the 6 cell lines, although we noticed different percentages of mice with detectable grafts (Supplementary Table 1a). These differences in the presence of grafts in the implanted mice could be due to technical or biological parameters. Although perinatal tolerization of transplanted human cells in immunocompetent mice has been controversial [20], we and others have previously demonstrated the long-term survival of neonatally engrafted hNPCs [16, 21, 22]. Rejection of cells xenografted at neonatal stages showed by other authors could be due to an excessive cell dosage, to the large diameter of the cannula, to mouse strain or to the hPSC line used. However, assessment of microglial and astroglial reactivity around the bulk of our surviving grafts showed no significant differences between CTR and HD cells (Supplementary Fig. 2). To verify whether possible immunological rejection—not detected by microglial and astroglial reactivity—could affect hNPC survival, we further tested hPSC survival in a genetically immunosuppressed mouse model, the *Rag2*^*−/−*^*.* We found low immunoreactivity surrounding the neonatally implanted hNPCs (Supplementary Fig. 3a, b), allowing the survival of similar numbers of human cells one month after grafting compared with WT mice (Supplementary Fig. 3c).

At 3 days PST, HD-60-derived hNPCs showed a higher proliferation rate (Supplementary Fig. 4a) that resulted in an increased number of cells at 1 month PST, compared to CTR-33-derived hNPCs (Supplementary Fig. 4b). This early alteration was already present in HD-hNPCs at 16 DIV, just before transplantation (% Ki67^+^/DAPI^+^ cells: CTR, 52 ± 3.72%; HD, 63 ± 3.75%. Unpaired *t* test, two-tailed, *P* = 0.027). We did not observe differences in the % of Ki67^+^ cells between the different CTR cell lines, nor between the HD lines we used in this study. In addition, no differences in the number of Ebf^+^ or Dlx^+^ positive hNPCs were observed at 16 DIV between CTR and HD cell lines (Introna et al., *in preparation*). Because recent work has reported the existence of a persistent mitotically active cyclin D1^+^ progenitor population in HD iPSCs during differentiation [23], we further examined cyclin D1 expression in our cells after implantation. We found that the higher proliferation rate correlated with an increased expression of cyclin D1 at 3 days PST, which persisted in subsets of HD-60-hNPCs up to 1 month PST (Supplementary Fig. 4b, c). Proliferating cells decreased over time until 3 months PST, when they were barely detected. Accordingly, we did not observe tumour formation at any of the time points examined.

**Fig. 1:**
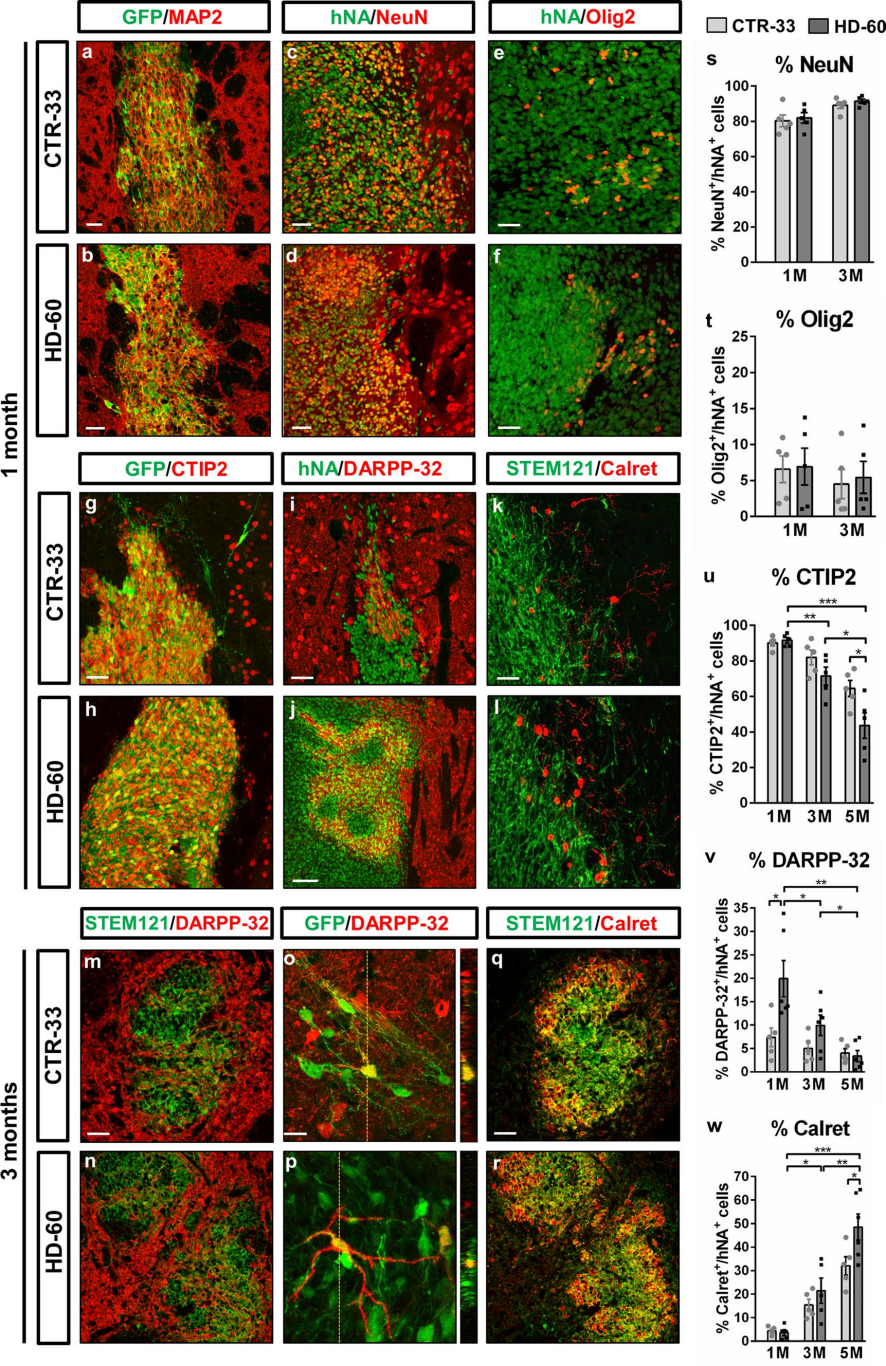
Neonatally engrafted control and HD patient-derived human neural progenitor cells differentiate into striatal neurons. **a**–**l** Striatal coronal sections from CTR-33 and HD-60 chimeric brains at 1 month PST immuno-labelled for GFP, hNA or STEM121 (green) and MAP2 (**a**, **b**), NeuN (**c**, **d**), Olig2 (**e**, **f**), CTIP2 (**g**, **h**), DARPP-32 (**i**, **j**) or Calretinin (Calret) (**k**, **l**) (red). **m**–**r** Striatal coronal sections from CTR-33 and HD-60 chimeric brains at 3 months PST immunolabelled for STEM121 or GFP (green) and DARPP-32 (**m**–**r**) or Calret (**q**, **r**) (red). **s**–**w** Histograms representing the percentage of hNA^+^ cells expressing NeuN (**s**), Olig2 (**t**), CTIP2 (**u**), DARPP-32 (**v**) and Calret (**w**) in CTR-33 and HD-60 chimeric brains at 1, 3 and 5 months PST. *M*, months. Scale bars 20 µm in **o**; 50 µm in **a**–**e**, **g**, **i**, **k**, **m**, **q**; 100 µm in **j**. Data are expressed as mean ± SEM. **s**, **t*** n* = 5 mice; Unpaired t-test, two-tailed, non-significant. **u** *n* = 5 mice; Unpaired t-test, two-tailed, *P* = 0.0465 (CTR 5 M vs HD 5 M), *P* = 0.0051 (HD 1 M vs HD 3 M),* P* = 0.0133 (HD 3 M vs HD 5 M), *P* = 0.0002 (HD 1 M vs HD 5 M). **v** *n* = 5 mice; Unpaired t-test, two-tailed, *P* = 0.0273 (CTR 1 M vs HD 1 M), *P* = 0.0489 (HD 1 M vs HD 3 M), *P* = 0.0254 (HD 3 M vs HD 5 M),* P* = 0.0022 (HD 1 M vs HD 5 M). **w ***n*  = 5 mice; Unpaired t-test, two-tailed, *P* = 0.0451 (CTR 5 M vs HD 5 M),* P* = 0.0117 (HD 1 M vs HD 3 M),* P* = 0.0072 (HD 3 M vs HD 5 M),* P* < 0.0001 (HD 1 M vs HD 5 M). See also Supplementary Table 2

At 1 month PST, ~ 80% of human cells already expressed markers of mature neurons (NeuN and MAP2), in a similar manner for both CTR-33 and HD-60 cell lines (Fig. 1a–d, s and Supplementary Table 2). ~ 90% of engrafted cells were CTIP2^+^ (Fig. 1g, h, u) including a DARPP-32^+^ subpopulation (Fig. 1i, j, v), indicative of MSN identity. Remarkably, HD-60 cells exhibited increased DARPP-32 expression compared to CTR-33 cells (20% vs 7%) at 1 month PST (Fig. 1v). By 3 months PST, human cells disseminated throughout the striatum showing increased branching, and some DARPP-32^+^ cells displayed a MSN-like morphology (Fig. 1m-p). Alternative cell fates included Olig2^+^ oligodendrocytes (Fig. 1e, f, t) and Calretinin (Calret)^+^ interneurons (Fig. 1k, l, q, r, w), which showed slower maturation and were mainly located at the edges of the graft. We also observed engrafted cells at 3 and 5 months PST in animals transplanted with GEN-019-, GEN-020-, CTR-2190 and HD-2174-derived hNPCs (Supplementary Figs. 5 and 7), reproducing the high expression of CTIP2 observed in HD-60 and CTR-33. The increased cell proliferation detected in HD cell lines resulted in increased graft volumes with respect to CTR hNPC grafts (Supplementary Table 1b). However, considerable variation was observed between HD transplants, being the HD-60 and GEN-20 the hNPCs that showed bigger grafts (Supplementary Fig. 5). Neither CTR-33 nor HD-60 cells colocalized with markers of GABAergic striatal interneuron subtypes, including Parvalbumin, Neuropeptide Y and Tyrosine hydroxylase (Supplementary Fig. 6).

### Human striatal neurons send axonal projections to MSN targets and establish synaptic connexions within the mouse basal ganglia circuitry

**Fig. 2:**
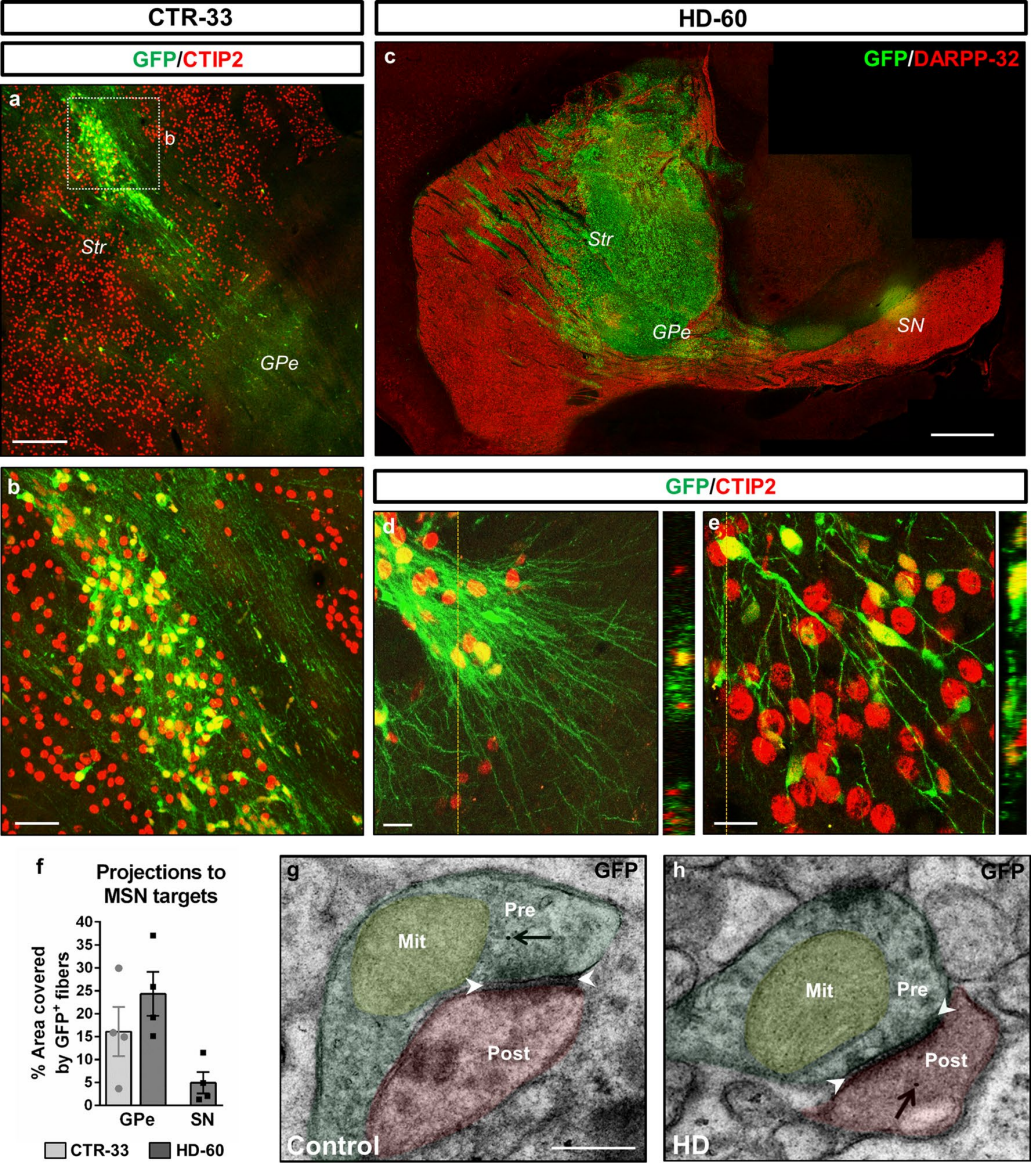
Human striatal neurons send axonal projections towards MSN targets and establish synapses within the mouse basal ganglia circuitry. **a**–**e** Sagittal sections from CTR-33 and HD-60 chimeric brains at 3 months PST, immuno-labelled for GFP (green), and CTIP2 or DARPP-32 (red). **f** Histogram representing the percentage of striatopallidal and nigral areas covered by GFP^+^ human fibres. **g**, **h** Ultra-thin immunogold TEM sections showing GFP^+^ human neurites establishing symmetric inhibitory synapses with host striatal cells. Black arrows point to human-specific gold nanoparticles and white arrowheads delimit the synaptic cleft of symmetric synapses. *Str* striatum, *GPe* external globus pallidus, *SN* substantia nigra, *Pre* presynaptic terminal, *Post* postsynaptic terminal, *Mit* mitochondria. Scale bars 0.5 µm in **g**; 20 µm in **d**, **e**; 50 µm in **b**; 200 µm in **a**; 1 mm in **c**. Data are expressed as mean ± SEM. *n* = 4 mice

Immunohistochemistry (IHC) on sagittal sections of chimeric mouse brains revealed that CTIP2^+^ human striatal neurons sent numerous GFP^+^ axonal projections towards the external *globus pallidus* (GPe), and few of them towards the *substantia nigra* (SN), two well-known MSN targets (Fig. 2a–f). Noteworthy, only HD-60 cells projected to both brain regions, because no CTR cell-derived projections were found in the SN. This might be due to the bigger size of the HD-60 graft, as a result of higher hNPC proliferation and spreading. Furthermore, transmission electron microscopy (TEM) combined with GFP immunogold labelling allowed the identification of inhibitory symmetric synapses between human and mouse cells (Fig. 2g, h), suggesting graft-to-host functional connectivity. Altogether, these data indicate that most transplanted hNPCs differentiate into striatal neurons, which send axonal projections towards their natural targets of the host basal ganglia circuitry.

### Selective degeneration and loss of HD human MSNs and host striatal tissue

**Fig. 3:**
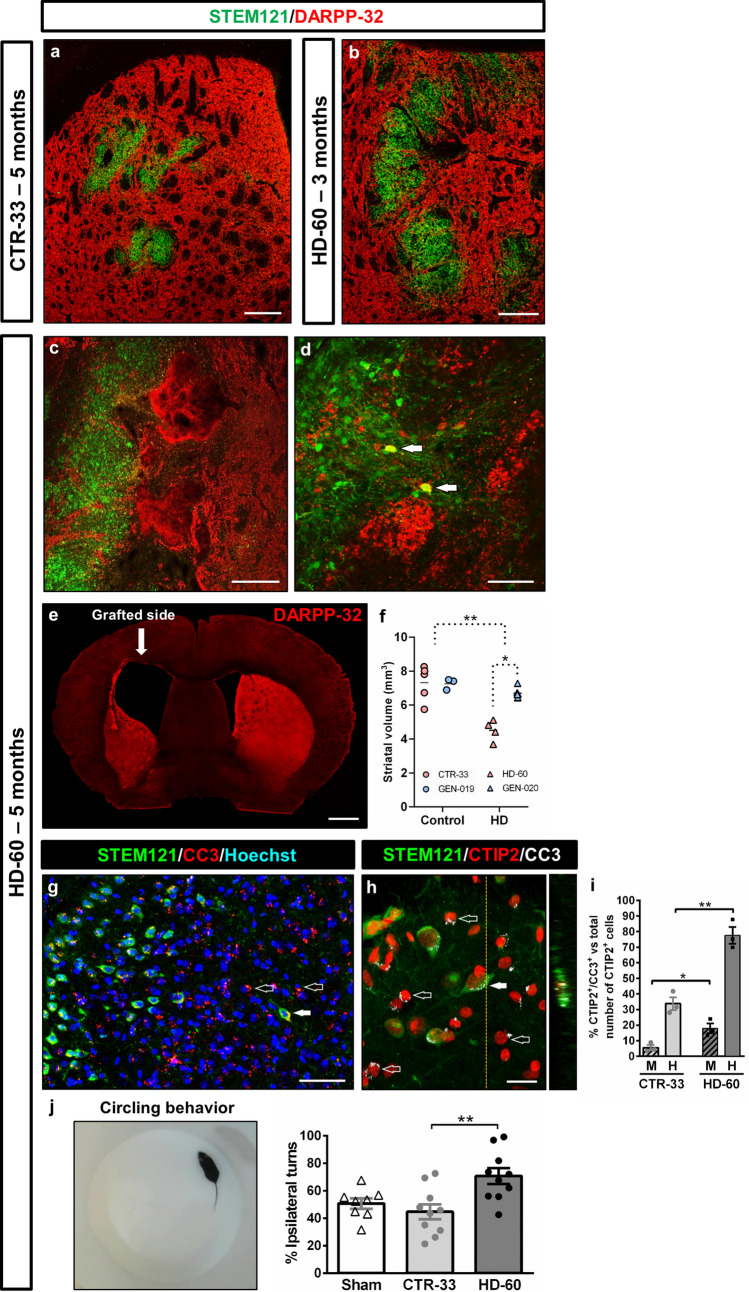
Selective cell death of engrafted HD human MSNs and degeneration of the host striatum alters mouse behaviour at 5 months PST. **a**–**d** Striatal coronal sections from CTR-33 and HD-60 chimeric brains immuno-labelled for STEM121 (green) and DARPP-32 (red). **d** Arrows point to DARPP-32^+^ HD-60 cells surrounded by degenerating mouse striatal tissue. **e** Low-magnification coronal view of a HD-60 chimeric brain at 5 months PST labelled for DARPP-32, exhibiting a dramatic increase in ventricular volume. **f** Histogram representing the assessment of striatal volume in CTR and HD chimeric mouse brains at 5 months PST. **g**, **h** Striatal coronal sections from HD-60 chimeric brains at 5 months PST immunolabelled for STEM121, CTIP2 and cleaved caspase-3 (CC3). Filled arrows point to apoptotic human striatal neurons and empty arrows point to apoptotic endogenous mouse cells. **i** Graph showing the percentage of apoptotic striatal neurons amongst mouse (M) and human (H) cells, in both CTR-33 and HD-60 chimeric mice. **j** Amphetamine-induced circling behavioural test performed with Sham, CTR-33 and HD-60 chimeric mice at 5 months PST. *M* mouse, *H* human. Scale bars 20 µm in **h**; 50 µm in **d**, **g**; 200 µm in **a**–**c**; 1 mm in **e**. Data are expressed as mean ± SEM. **f** *n* = 8 Control [CTR-33 (*n* = 5, pink), GEN-019 (*n* = 3, blue)] and *n* = 10 HD [HD-60 (*n* = 4, pink), GEN-020 (*n* = 6, blue)] mice; Unpaired t-test, two-tailed, *P* = 0.0093 (Control vs HD),* P* = 0.0490 (HD-60 vs GEN-020). **i** *n* = 3; Unpaired t-test, two-tailed, *P* = 0.0251 (CTR-33 M vs HD-60 M), *P* = 0.0029 (CTR-33 H vs HD-60 H). **j** *n* = 8 Sham, *n* = 10 CTR-33 and *n* = 10 HD-60 mice; One-way ANOVA, Tukey’s multiple comparisons test, *P* = 0.0035 (CTR-33 vs HD-60)

Robust MSN loss is a crucial HD hallmark that is lacking in the existing mouse models. Transplanted CTR-33- (Fig. 3a), GEN-019- and CTR-2190-hNPCs (Supplementary Fig. 7) survived up to 5 months PST, with no signs of degeneration and without affecting mouse host striatal cells. Conversely, engrafted HD-60-hNPCs reached their peak of MSN differentiation at 1 month PST, with 92% of CTIP2^+^ cells and 20% of DARPP-32^+^ cells. But from that time onwards they showed a progressive decrease, with 71% and 10% of cells expressing CTIP2 and DARPP-32, respectively, at 3 months PST, and only 44% and 3% at 5 months PST (Fig. 1u, v and Supplementary Table 2). Overall, there was a 52% decrease of CTIP2 expression and a 75% reduction in DARPP32^+^ human MSNs. Unlike HD-60 MSNs, HD Calret^+^ interneurons did not undergo degeneration, but instead maintained and even increased their relative number over time (Fig. 1w and Supplementary Table 2).

Most strikingly, when compared to the 3 months PST time point of HD-60 cells (Fig. 3b) or GEN-020 cells (Supplementary Fig. 5b), the host striatum exhibited clear signs of degeneration in the vicinity of engrafted HD cells at 5 months PST (Fig. 3c, d and Supplementary Fig. 8b). There was a 20% loss of striatal tissue (Fig. 3f), which in the case of HD-60 chimeric mice rose to 38% and was accompanied by an increase of ventricular volume similar to that seen in end-stage HD human post-mortem brains (Fig. 3e). Therefore, striatal neurodegeneration was more prominent in mice implanted with HD-60-hNPCs (38%) than in animals transplanted with GEN-020-hNPCs (8%), maybe due to the higher number of CAG repeats (60 CAG vs 48 CAG repeats, respectively). Accordingly, both human and mouse CTIP2^+^ cells expressed apoptotic markers (Fig. 3g–i and Supplementary Fig. 8c, d), which were much less frequent in controls (Fig. 3i and Supplementary Fig. 9). Amphetamine-induced circling behavioural test confirmed unilateral striatal degeneration in HD-60 chimeric mice (Fig. 3j), as showed by the increased number of ipsilateral turns towards the transplanted brain hemisphere (Supplementary Video 1) compared to CTR-33 chimeric mice (Supplementary Video 2). Hence, HD chimeric mice show selective degeneration and loss of human MSNs, with deleterious effects on neighbouring mouse striatal MSNs.

### HD human neurons show progressive ultrastructural alterations

**Fig. 4:**
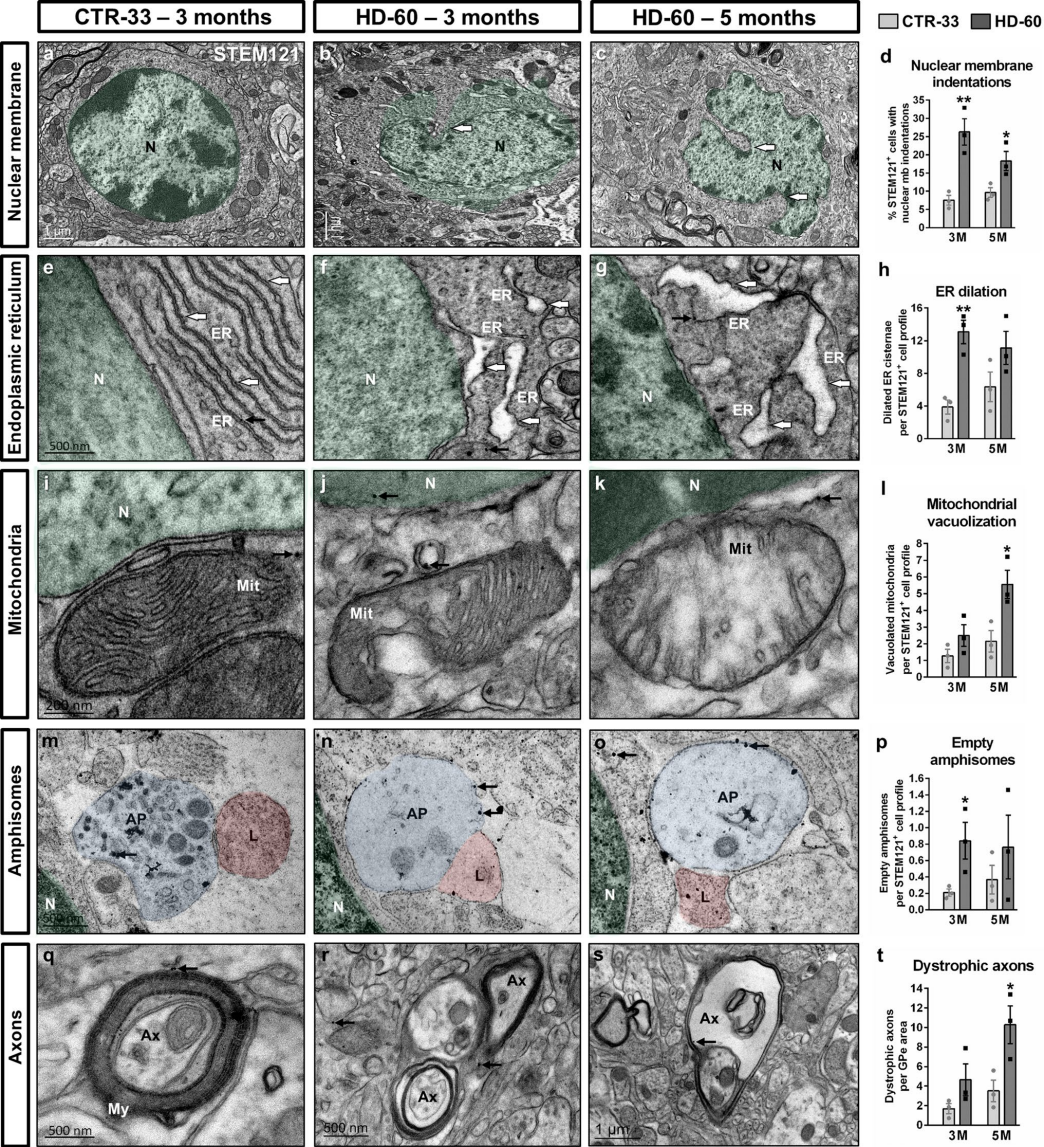
Transplanted HD human cells show progressive ultrastructural alterations from 3 months PST onwards. Ultra-thin striatal sections from CTR-33 and HD-60 chimeric brains immunogold-labelled for STEM121 and analysed by TEM at 3 and 5 months PST. **a**–**d** Illustrative TEM images and histogram (**d**) showing the percentage of human transplanted cells with nuclear membrane indentations. **e**–**h** Illustrative TEM images and histogram (**h**) showing the number of dilated endoplasmic reticulum cisternae per cell. **i**–**l** Illustrative TEM images and histogram (**l**) showing the number of vacuolated mitochondria per cell. **m**–**p** Illustrative TEM images and histogram (**p**) showing the number of empty amphisomes per cell. **q**–**t** Illustrative TEM images and histogram (**t**) showing the number of dystrophic axons per external globus pallidus (GPe) area. Small black arrows point to human-specific gold nanoparticles. GPe area in **t**: 2.34 × 10^–4^ mm^2^. *N* nucleus, *ER* endoplasmic reticulum, *Mit* mitochondria, *AP* amphisome, *L* lysosome, *Ax* axon, *My* myelin, *M* months. Data are expressed as mean ± SEM. **d** *n* = 3 mice; Unpaired t-test, two-tailed, *P* = 0.0083 (3 M), *P* = 0.0419 (5 M). **h ***n*  = 3 mice; Unpaired t-test, two-tailed, *P* = 0.0052 (3 M), *P* = 0.1546 (5 M). **l ***n*  = 3 mice; Unpaired t-test, two-tailed, *P* = 0.1828 (3 M), *P* = 0.0314 (5 M). **p ***n*  = 3 mice; Unpaired t-test, two-tailed, *P* = 0.0498 (3 M), *P* = 0.4037 (5 M). **t ***n* =  3 mice; Unpaired t-test, two-tailed, *P* = 0.1612 (3 M), *P* = 0.0381 (5 M)

To analyse the progressive dysfunction of HD human cells at the finest structural level, we examined HD-60 chimeric brains by TEM immunogold at 3 and 5 months PST. HD cells underwent substantial morphological changes from 3 months onwards, including nuclear membrane indentations (Fig. 4a–d), dilated ER cisternae (Fig. 4e–h) and abnormally swollen mitochondria with sparse cristae (Fig. 4i–l). TEM also revealed the existence of mostly empty amphisome-like structures in HD cells (Fig. 4m–p), indicating altered autophagy. These remarkable alterations observed at the striatal level correlated with the presence of dystrophic axons at the GPe (Fig. 4q–t). Taken together, ultrastructural defects found in HD-60 human cells are consistent with the progressive neurodegeneration observed by IHC.

### Gradual appearance of soluble and aggregated mHTT in key cellular organelles and axons

**Fig. 5:**
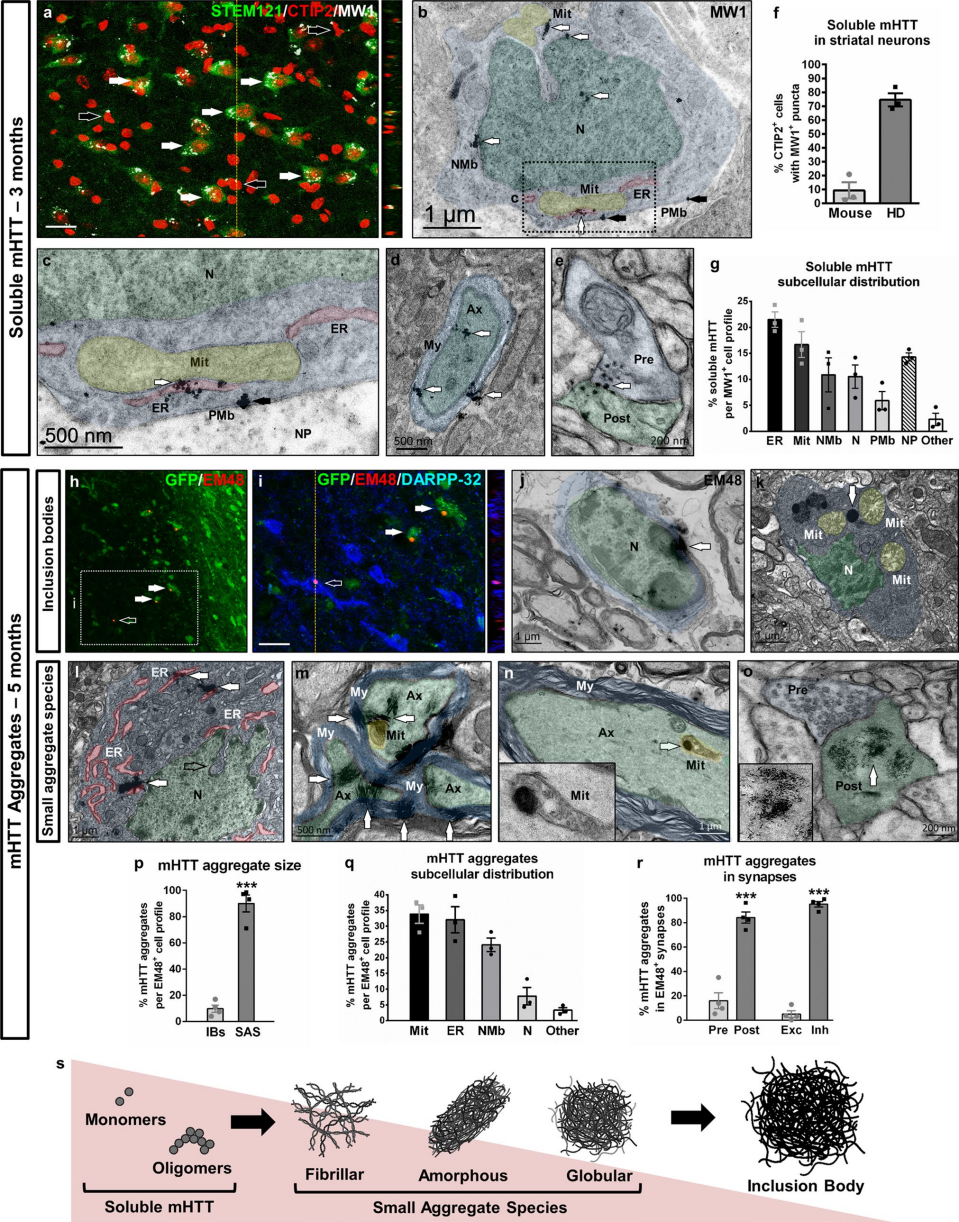
Gradual appearance of soluble and aggregated mHTT in human striatal neurons. **a** Illustrative striatal coronal section from a HD-60 chimeric brain at 3 months PST immuno-labelled for STEM121 (green), CTIP2 (red) and MW1 (white). White arrows point to MW1^+^ puncta inside STEM121^+^/CTIP2^+^ human striatal neurons, whilst black arrows point to MW1^+^ puncta in STEM121^−^/CTIP2^+^ mouse striatal neurons. **b**–**e** Ultra-thin striatal sections from HD-60 chimeric brains immunogold-labelled for MW1 and analysed by TEM at 3 months PST. White arrows in **b** and **c** point to cytoplasmic MW1^+^ mHTT monomers/oligomers, whilst black arrows point to soluble mHTT located at the plasma membrane boundary. **d** and **e** illustrate the presence of soluble mHTT in myelinated axons and synaptic terminals, respectively. **f** Histogram representing the percentage of mouse and human CTIP2^+^ cells containing MW1^+^ puncta. **g** Histogram representing the subcellular distribution of soluble mHTT monomers/oligomers. **h**, **i** Striatal coronal sections of HD-60 chimeric brains at 5 months PST immunolabelled for GFP (green), EM48 (red) and DARPP-32 (blue). **j**–**o** Ultra-thin striatal sections from HD-60 chimeric brains immunogold-labelled for EM48 and analysed by TEM at 5 months PST, depicting inclusion bodies (**j**, **k**) and small aggregate species (**l**–**o**). **p**–**r** Histograms representing mHTT aggregate size (**p**), subcellular distribution (**q**) and location in synapses (**r**). **s** Summary of the different mHTT species found during the progressive degeneration of HD-hNPCs. *N* nucleus, *NMb* nuclear membrane, *PMb* plasma membrane, *ER* endoplasmic reticulum, *Mit* mitochondria, *Ax* axon, My myelin, *Pre* presynaptic terminal, *Post* postsynaptic terminal, *NP* neuropil, *IBs* inclusion bodies, *SAS* small aggregate species, *Exc* excitatory synapses, *Inh* inhibitory synapses. Scale bars 20 µm in **a**, **i**. Data are expressed as mean ± SEM. **f**, **g** *n* = 3 mice; **p** *n* = 4 mice; Unpaired t-test; two-tailed, *P* < 0.0001. **r ***n*  = 4 mice; Unpaired t-test; two-tailed, *P* < 0.0001 (Presynaptic vs Postsynaptic),* P* < 0.0001 (Excitatory vs Inhibitory)

We next investigated whether morphological alterations of subcellular organelles in HD human cells correlated with the gradual appearance of mHTT species, the main neuropathological hallmark of HD. We first examined if soluble non-aggregated forms of mHTT could be detected before overt degeneration, when neuronal dysfunction starts to manifest. To this aim we performed IHC with MW1 antibody, a marker of mHTT oligomers that may also detect monomers [24], which showed positive puncta staining in most CTIP2^+^ human cells (~ 70%) at 3 months PST (Fig. 5a, f and Supplementary Fig. 10). A more detailed examination by means of TEM immunogold determined that soluble mHTT primarily interacted with ER and mitochondria, and to a lesser extent with nuclear membrane and nucleus (Fig. 5b, c, g). Importantly, soluble mHTT was also found in myelinated axons and synaptic terminals of inhibitory symmetric synapses at the GPe level (Fig. 5d, e).

In order to assess the presence of insoluble mHTT aggregated forms in transplanted HD cells during overt neurodegeneration, we performed both IHC and TEM immunogold with EM48 antibody at 5 months PST. By IHC we only detected a small subpopulation of human cells containing mHTT aggregates in HD-60 chimeric mice (4.14% ± 0.64, *n* = 5), corresponding to inclusion bodies (IBs) (Fig. 5h, i), but we could not detect mHTT aggregates in GEN-020 chimeric mice. Nevertheless, TEM analysis revealed that the IBs found in HD-60 chimeric brains corresponded only to 9% of total EM48^+^ aggregated forms of mHTT (Fig. 5j, k, p), because small aggregate species (SAS) (≤ 400 nm) were much more abundant (Fig. 5l–o, p). Amongst SAS, we identified three different morphologies (summarised in Fig. 5s): fibrillar (Fig. 5m, o), amorphous (Fig. 5l) and globular (Fig. 5n). The subcellular distribution of mHTT SAS was similar to that of soluble forms (Fig. 5q), with a preferential association to mitochondria (Fig. 5k, n), ER (Fig. 5l) and nuclear membrane (Fig. 5j). Consistently, SAS were also found in myelinated axons (Fig. 5m, n) and synaptic terminals of inhibitory symmetric synapses at the GPe level, mainly at postsynaptic sites (Fig. 5o, r). In addition, STEM121^−^ axons at the striato-pallidal level contained bundles of mHTT-like fibrils in close proximity to mitochondria (Supplementary Fig. 11). Overall, this spatio-temporal morphometric characterisation of mHTT enabled us to determine the timing, structure and subcellular localization of mHTT species in HD human cells in vivo.

### Human-to-mouse transfer of mHTT correlates with increased exosomal secretory pathway

**Fig. 6:**
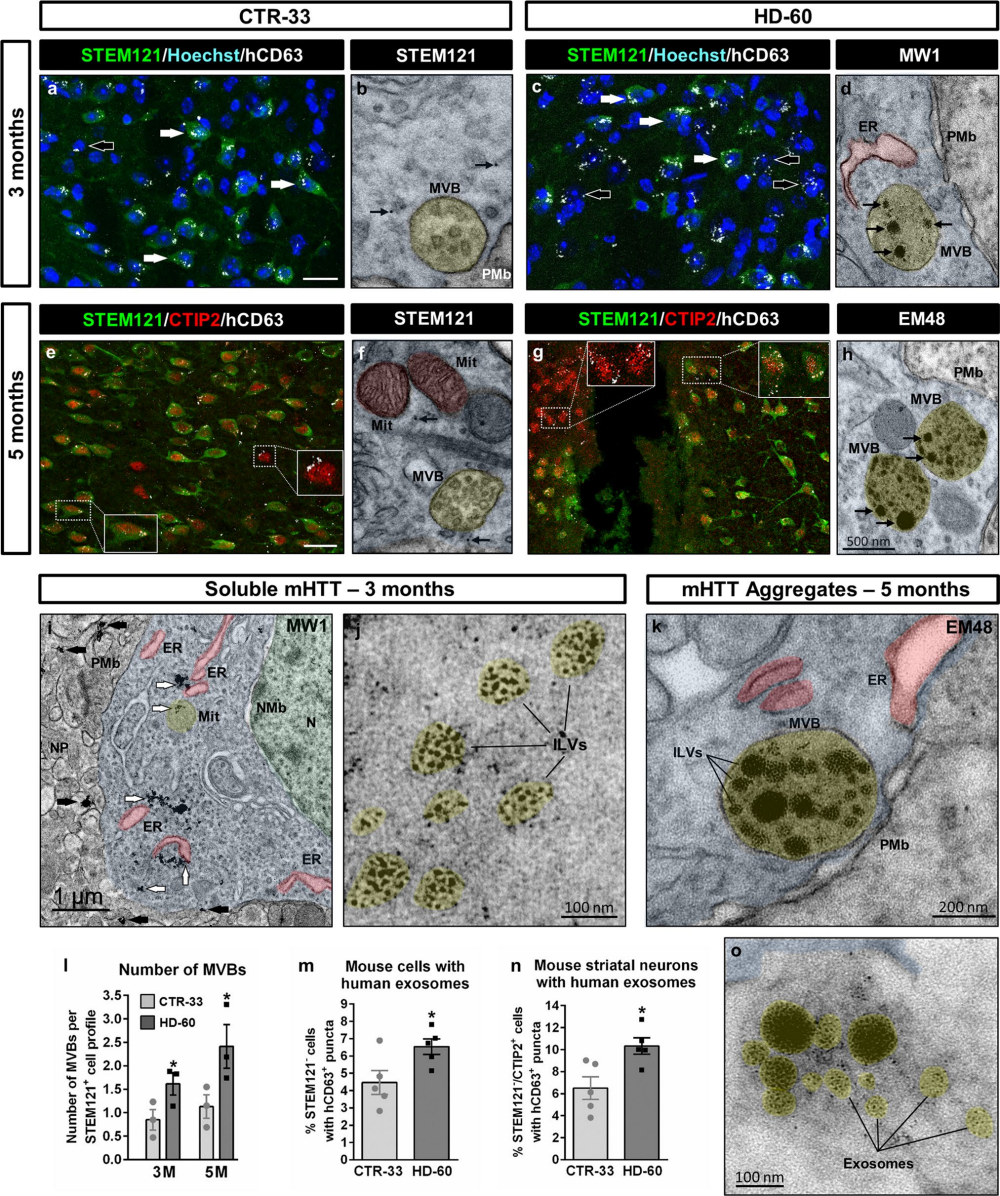
Upregulation of the exosomal secretory pathway in human striatal neurons. **a**, **c** Striatal coronal sections from CTR-33 and HD-60 chimeric brains at 3 months PST immuno-labelled for STEM121 (green) and hCD63 (white). White arrows point to hCD63^+^ puncta inside STEM121^+^ human cells, whilst black arrows point to hCD63^+^ puncta in close association with STEM121^−^ mouse cells. **e**, **g** Striatal coronal sections from CTR-33 and HD-60 chimeric brains at 5 months PST immunolabelled for STEM121 (green), CTIP2 (red) and hCD63 (white). Note the presence of hCD63^+^ puncta in both STEM121^+^/CTIP2^+^ human striatal neurons and STEM121^−^/CTIP2^+^ mouse striatal neurons (insets). **b**, **f** Ultra-thin sections from CTR-33 chimeric brains immunogold-labelled for STEM121 (black arrows) and analysed by TEM, illustrating the presence of MVBs. **d**, **h** Ultra-thin sections from HD-60 chimeric brains immunogold-labelled for MW1 or EM48 (black arrows) and analysed by TEM, illustrating the presence of mHTT inside intraluminal vesicles of a growing number of MVBs. **i**, **j** Illustrative ultra-thin sections from HD-60 chimeric brains at 3 months PST immunogold-labelled for MW1 and analysed by TEM, exhibiting intracellular (white arrows) and extracellular (black arrows) soluble mHTT (**i**), as well as MW1^+^ particles inside intraluminal vesicles (**j**). **k**, **o** Ultra-thin sections from HD-60 chimeric brains immunogold-labelled for EM48 and analysed by TEM, illustrating a MVB containing mHTT that is about to fuse with the plasma membrane (**k**) and another one that has just fused (**o**), secreting its content as exosomes. **l**–**n** Histograms representing the number of MVBs per transplanted human cell at 3 and 5 months PST (**l**), the percentage of mouse cells with human exosomes at 3 months PST (**m**) and the percentage of mouse striatal neurons with human exosomes at 5 months PST (**n**).* N* nucleus, *NMb* nuclear membrane, *Mit* mitochondria, *ER* endoplasmic reticulum, *PMb* plasma membrane, *NP* neuropil, *MVB* multivesicular body, *ILVs* intraluminal vesicles. Scale bar 20 µm in **a**, **e**. Data are expressed as mean ± SEM. **l** *n* = 3 mice; Unpaired t-test, two-tailed, *P* = 0.0406 (3 months),* P* = 0.0231 (5 months). **m ***n*  = 4 mice; Unpaired t-test, two-tailed, *P* = 0.0365. **n ***n*  = 4 mice; Unpaired t-test, two-tailed, *P* = 0.0165

The presence of MW1^+^ puncta in a subset of CTIP2^+^ mouse striatal neurons (~ 10%) at 3 months PST (Fig. 5a, f) and even EM48^+^ aggregates in scarce GFP^−^/DARPP-32^+^ cells at 5 months PST (Fig. 5i), indicated human-to-mouse transfer of mHTT. In addition, TEM studies revealed that a considerable number of mHTT oligomers/monomers (~ 20%) were located either at the plasma membrane or outside the cell body (Figs. 5b, c, g and 6i), further suggesting ongoing secretion of soluble mHTT. Interestingly, these mHTT forms exhibited a rather loose appearance within the cytoplasm, but they were more densely packed when located at the plasma membrane boundary or extracellularly (Figs. 5c and 6i), supporting the notion that they could be secreted inside EVs. To investigate if the exosomal secretory pathway was upregulated in HD human cells, we examined by IHC the expression pattern of a human-specific CD63 antibody (hCD63), which labels both intraluminal vesicles of multi-vesicular bodies (MVBs) and human exosomes [25]. At 3 months PST, a higher number of mouse endogenous cells had incorporated hCD63^+^ puncta in HD-60 chimeric mice, suggesting increased exosomal secretion by HD human cells (Fig. 6a, c, m). Remarkably, at 5 months PST a higher proportion of CTIP2^+^ mouse striatal neurons contained hCD63^+^ puncta in HD-60 chimeric mice, particularly in the vicinity of areas undergoing degeneration (Fig. 6e, g, n). Quantification of the number of MVBs by TEM revealed an increase in HD-60 human cells at both 3 and 5 months PST (Fig. 6b, d, f, h, l), likely reflecting an upregulation of the exosomal secretory pathway. Most importantly, we found MW1^+^ and EM48^+^ particles within intraluminal vesicles of MVBs (Fig. 6d, h, j), which could fuse with the plasma membrane (Fig. 6k) and be released as exosomes (Fig. 6o). Noteworthy, MVBs containing mHTT were also occasionally detected at the presynaptic terminals of inhibitory symmetric synapses (Supplementary Fig. 12a, b), and EM48^+^ exosome-like vesicles were found in sparse dead cells within the HD-60 chimeric striatum (Supplementary Fig. 12c). Altogether, these findings strongly suggest that mHTT can be transferred from transplanted human neurons to endogenous striatal neurons through exosomes.

### HD human neuronal cells secrete extracellular vesicles that propagate toxic soluble mHTT to mouse striatal neurons

**Fig. 7:**
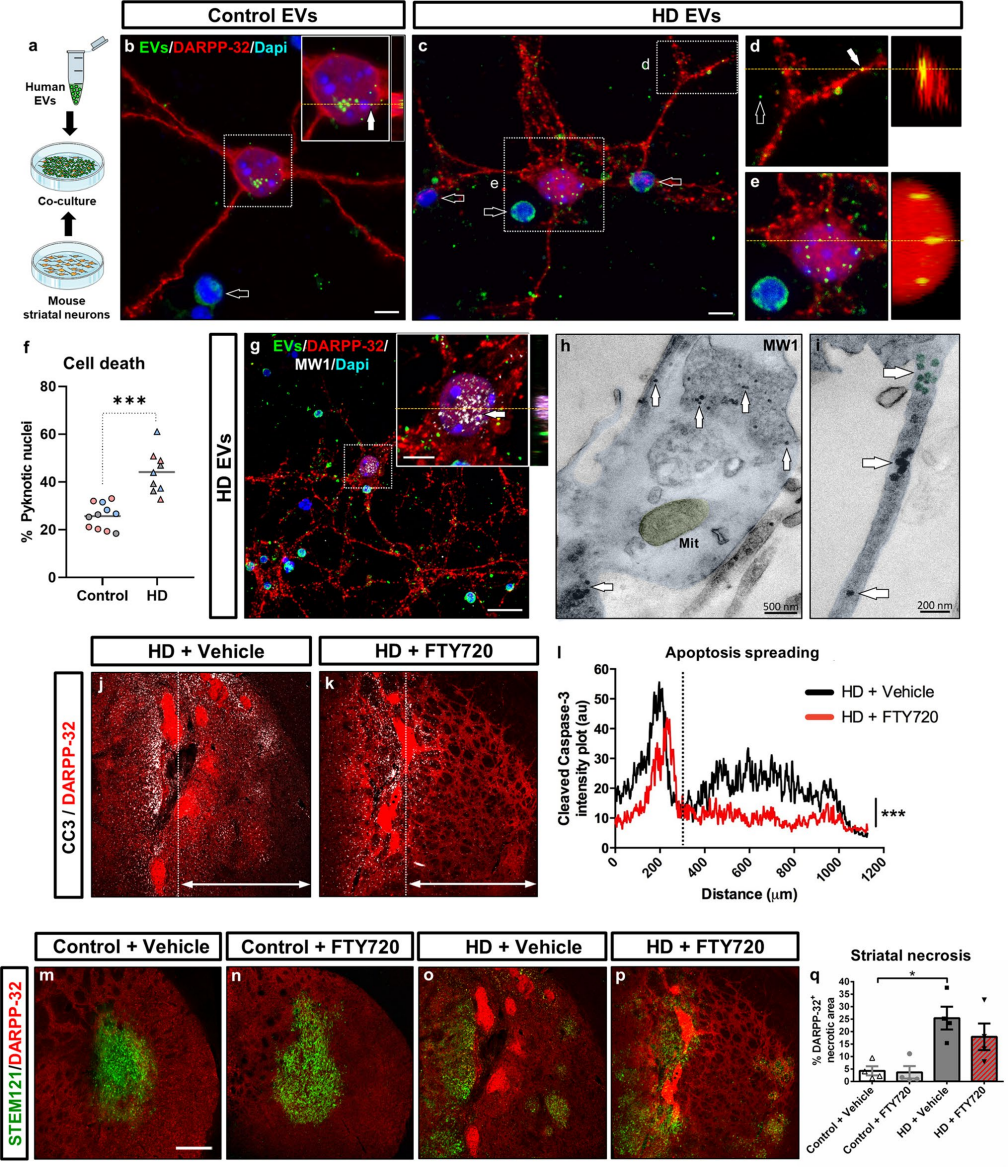
HD human neuronal cells secrete extracellular vesicles that propagate toxic soluble mHTT to mouse striatal neurons. **a** Schematic representation of the co-culture experiment. **b**–**e** Co-culture of fluorescently labelled human EVs (green) (filled arrows) isolated from CTR and HD-hNPCs at 16 DIV with DARPP-32^+^ mouse primary striatal neurons (red), for 24 h at a 2:1 ratio (EV-donor cells: EV-recipient cells). Empty arrows in **b**, **c** point to pyknotic nuclei. **f** Histogram showing the number of pyknotic nuclei in CTR and HD co-cultures. **g**–**i** Immunocytochemical and TEM immunogold-labelling for MW1 of mouse striatal neurons co-cultured with human HD EVs, showing the presence of MW1^+^ monomers/oligomers (white arrows) inside mouse cells. **j**, **k** Striatal coronal sections from HD-60 chimeric mice treated with either vehicle or FTY720 at 5 months PST, and immuno-labelled for DARPP-32 (red) and cleaved caspase-3 (white). **l** Quantification of apoptosis spreading from the bulk of the graft (dotted line in **j** and **k**), by analysing cleaved caspase-3 intensity plot profile. **m**–**p** Striatal coronal sections from CTR-33 and HD-60 chimeric mice treated with either vehicle or FTY720 at 5 months PST, and immuno-labelled for STEM121 (green) and DARPP-32 (red). **q** Histogram representing the degree of striatal necrosis in treated vs non-treated chimeric mice. *EVs* extracellular vesicles, *Mit* mitochondria. Scale bars 5 µm in **b**, **c**; 20 µm in **g**; 200 µm in **m**; Data are expressed as mean ± SEM. **f** *n* = 11 Control [CTR-33 (*n* = 5, pink), CTR-2190 (*n* = 3, grey), GEN-019 (*n* = 3, blue)] and *n* = 9 HD [HD-60 (*n* = 3, pink), HD-2174 (*n* = 3, grey), GEN-020 (*n* = 3, blue)] co-cultures; Unpaired t-test, two-tailed, *P* < 0.0001. **l** *n* = 4 mice; Kolmogorov–Smirnov t-test, two-tailed, *P* < 0.0001. **q** *n* = 4 mice; Two-way ANOVA, Tukey’s multiple comparisons test,* P* = 0.0324 (Control + Vehicle vs HD + Vehicle), *P* = 0.6367 (HD + Vehicle vs HD + FTY720)

To delve deeper into the mechanism of exosome-mediated mHTT propagation and its potential deleterious effects on mouse striatal neurons, we next isolated EVs from the conditioned culture medium of 3 HD-derived hNPCs lines (HD-60, GEN-020, HD-2174) and 3 CTR-derived hNPCs lines (CTR-33, GEN-019 and CTR-2190) at 16 DIV by SEC. CD63^+^ and CD81^+^ EV fractions were identified in both types of cells (Supplementary Fig. 13a, b). Human EVs isolated at 16 DIV were fluorescently labelled with CFSE and co-cultured for 24 h with mouse primary striatal neurons at a 2:1 ratio (EV-donor cells: EV-recipient cells) (Fig. 7a). Both CTR- and HD-released EVs were taken-up by DARPP-32^+^ mouse MSNs, but HD EVs induced higher cell death, as shown by the increased number of pyknotic nuclei (Fig. 7b–f and Supplementary Fig. 13c, d). This finding prompted us to identify the neurotoxic cargo inside HD EVs. Immunocytochemistry and TEM immunogold analysis revealed the presence of MW1^+^ soluble mHTT species (Fig. 7g–i and Supplementary Fig. 13c, d), but not EM48^+^ aggregates, within EVs and mouse striatal neurons. Because MW1 antibody detects mainly oligomeric forms, we performed additional TEM immunogold labelling with 3B5H10 antibody, which preferentially recognises mHTT monomers [26]. Remarkably, 3B5H10^+^ monomers were found encapsulated within HD EVs, fusing with the cell membrane and interacting with mitochondria inside mouse striatal neurons (Supplementary Fig. 14). Noteworthy, both MW1 and 3B5H10 antibodies have been reported to detect the mHTT species that best predict neurodegeneration [26, 27]. Based on these data, we conclude that HD neuronal cells secrete EVs carrying soluble mHTT monomers and oligomers, which can be internalised by mouse MSNs progressively seeding pathology and triggering cell death.

In order to investigate in vivo whether pharmacological inhibition of the exosomal secretory pathway could prevent the striatal degeneration observed in HD chimeric mice, we used the drug fingolimod (FTY720), a functional antagonist of sphingosine 1-phosphate receptors that blocks cargo sorting into exosomes [28, 29]. HD-60 and CTR-33 chimeric mice were treated with either FTY720 or vehicle solution from 1 to 3 months PST, and cell death was examined at 5 months PST. Cleaved caspase-3 staining revealed that FTY720 decreased the extent of apoptosis spreading from the bulk of the HD graft (Fig. 7j–l), likely reflecting a lowering of exosome-mediated mHTT propagation. Conversely, FTY720 did not significantly reduce striatal necrosis at the bulk of the HD graft (Fig. 7m–q), suggesting that treatment was specifically targeting mHTT spreading. We thus provide compelling evidence of the existence of an EV-mediated non-cell autonomous mechanism of mHTT toxicity.

## Discussion

We uncover here cell and non-cell autonomous mechanisms driving HD human pathogenesis that highlight the preponderant role of soluble mHTT as a trigger for neurodegeneration. First, by targeting essential cellular machinery, such as ER, mitochondria and nuclear membrane, and second, by spreading to neighbouring cells within EVs progressively seeding pathology.

We show increased proliferation and accelerated differentiation of HD-hNPCs at early stages, which match the initial striatal hypertrophy recently observed in child mHTT carriers [30]. van der Plas et al*.* reported that mHTT carriers exhibit a striking increase in striatal volume between 6 and 10 years of age, followed by a rapid decline in adolescence. This intriguing phenotype was particularly marked for children carrying more than 50 CAG repeats, with each incremental repeat being associated with greater hypertrophy and faster age-related striatal volume loss [30]. We observe a similar phenotype when transplanting HD-hNPCs, being more significant in cells with 60 CAG repeats (HD-60) than in cells with 48 CAG repeats (GEN-020). Therefore, we infer that we have recapitulated this aspect of early HD pathophysiology in our study. In addition, the higher MSN differentiation efficiency of HD-60 human cells could be due to a premature maturation of HD neuronal progenitors, as recently shown for cortical progenitors [31]. Altogether, our results support the hypothesis that HD begins with the abnormal development of neuronal subpopulations [13, 23, 31, 32].

We found a selective death of HD human MSNs, whereas cells differentiating into Calret^+^ interneurons were spared from neurodegeneration. The intriguing inverse correlation of survival between these two cell types throughout disease progression is human-specific, having been previously described in HD patients [33–35], but not in mouse models. Our results show that degeneration of HD human MSNs is accompanied by progressive axonal atrophy, manifested as dystrophic axons, disrupted myelin sheaths and synaptic neuronal terminals containing soluble mHTT and fibrils at the GPe level. These data are consistent with mHTT-associated axonal degeneration, one of the early pathological events in HD patients [36, 37] that particularly impinges on striatal neurons projecting to the GPe [3, 38]. The cortico-striatal pathway provides most of the excitatory glutamatergic input into the striatum and plays an important role in the development of the HD phenotype [39]. Nevertheless, in contrast to the work of Pecho-Vrieseling et al*.* [7], where transneuronal mHTT propagation needed proper connexions between cortical and striatal neurons, our human HD striatal cells followed progressive degeneration and mHTT dissemination without the input of cortical neurons.

We show the existence of soluble mHTT monomers and oligomers in the striatum of HD chimeric brains at a stage when early neuronal dysfunction starts to manifest. These forms primarily interacted with ER, mitochondria and nuclear membrane, inducing ultrastructural alterations (see schematic representation in Supplementary Fig. 15). These findings, together with compelling data in HD models and patients [40–45], implicate ER stress and mitochondrial dysfunction as important contributors to cellular pathology from the early stages of the disease. Because subcellular localization is critical for the effects of misfolded proteins, our data provide strong evidence that mHTT toxicity first arises as a result of pathological interactions of soluble forms with organelles that regulate key cellular processes, such as protein folding, autophagy and calcium homeostasis. Interestingly, we also found that localization of mHTT monomers/oligomers at the nuclear membrane correlated with abnormal indentations of the nuclear envelope, which have been described in post-mortem studies of HD human brains [46]. Our ultrastructural analysis revealed that the appearance of soluble mHTT species in HD patient’s cells was later followed by the development of small aggregate species, which were frequent on cells undergoing neurodegeneration. Nevertheless, only a small number of human striatal neurons developed large IBs, unlike transgenic HD mouse models and in agreement with clinical data [47, 48]. In the light of our findings and a growing body of work [26, 49–53], we postulate that mHTT toxicity is mainly related to the soluble forms of the misfolded protein, and that the formation of IBs in certain cells represents a protective mechanism to sequester other highly interacting mHTT species. Furthermore, the presence of soluble mHTT in the cerebrospinal fluid of HD patients has been identified as one of the earliest alterations over the course of the disease [27].

We also demonstrate that mHTT is targeted to MVBs for either autophagic degradation through the amphisome-lysosome pathway or secretion through exosomes. Interestingly, we detected a higher number of MVBs in HD human cells along with an accumulation of empty amphisomes and more extracellular exosomes, reflecting an up-regulation of the non-conventional secretory pathway at the expense of the classical degradation pathway. This switch assures the removal of toxic products from the cell, but it may favour the propagation of mHTT to neighbouring cells. In keeping with this idea, the striking degeneration of the healthy recipient mouse striatum following transplantation of HD-hNPCs points to a non-cell autonomous deleterious effect of HD cells on host tissue. Accordingly, both in vitro and in vivo analyses of the present study indicate that HD human cells transmit EVs containing toxic soluble mHTT monomers and oligomers to healthy mouse striatal neurons. Importantly, we show that blocking cargo sorting into exosomes with FTY720, a functional antagonist of sphingosine 1-phosphate receptors [28, 29], significantly decreases the spreading of apoptosis throughout the striatum of HD chimeric mice, reflecting lower mHTT transfer to mouse host cells. Conversely, FTY720 did not prevent the ultimate degeneration of transplanted HD-hNPCs, likely because the reduced mHTT exosomal secretion may result in a detrimental accumulation of toxic mHTT inside HD human neurons, accounting for their limited survival. Noteworthy, the injection of exosomes secreted by HD patient-derived fibroblasts with a much higher number of CAG repeats (143) induced similar mHTT toxicity in the healthy mouse striatum [8]. Further arguments supporting mHTT propagation come from the spreading of exogenous mHTT fibrils in mice [54] and the observation of mHTT aggregates within healthy foetal striatal allografts in HD patients [35, 55]. Altogether, our findings provide compelling evidence that mHTT exosome cargo, particularly soluble forms, participate in non-cell autonomous disease spreading.

Despite a multitude of therapeutic targets in preclinical models of neurodegenerative disorders, clinical translation has failed due to the difficulty of studying pathophysiology in living humans. We reveal here different aspects of HD human pathogenesis in vivo, allowing to elucidate the primary drivers of disease progression in patients. Our study sheds light into key questions that remained unclear in HD patients [39], such as the nature, structure and timing of mHTT species, their immediate cellular targets and their mechanism of cell-to-cell propagation. These findings will provide a novel conceptual framework for the development of effective therapeutic strategies for a devastating disorder with no cure to date.

## Supplementary Information

Below is the link to the electronic supplementary material.

Supplementary file1 (PDF 19342 KB)

Supplementary file2 (AVI 16104 KB)

Supplementary file3 (AVI 15796 KB)

## Data Availability

The data that support the findings of this study are included in this published article and its supplementary information files. Additional raw data from the corresponding author are available upon reasonable request.

## Abbreviations

BSA: Bovine serum albumin
Calret: Calretinin
CFSE: Carboxyfluorescein succinimidyl ester
CTR: Control
DIV: Days in vitro
ER: Endoplasmic reticulum
EV: Extracellular vesicle
HD: Huntington’s disease
hiPSC: Human-induced pluripotent stem cell
hNPC: Human neural progenitor cell
hCD63: Human CD63
hNA: Human nuclei antigen
IB: Inclusion body
IHC: Immunohistochemistry
mHTT: Mutant huntingtin
MSN: Medium spiny neuron
MVB: Multivesicular body
PBS: Phosphate-buffered saline
PFA: Paraformaldehyde
PST: Post-transplantation
SAS: Small aggregate species
SEC: Size-exclusion chromatography
SEM: Standard error of the mean
TEM: Transmission electron microscopy
WT: Wild-type
