# Supplementary material for: Soluble mutant huntingtin drives early human pathogenesis in Huntington’s disease
Source: Cell Mol Life Sci. 2023 Aug 3;80(8):238. doi: 10.1007/s00018-023-04882-w (PMC10400696; doi:10.1007/s00018-023-04882-w)
Supplement: Supplementary file 1 — Supplementary file1 (PDF 19342 KB) [file 18_2023_4882_MOESM1_ESM.pdf]

Supplementary information

Soluble mutant huntingtin drives early human pathogenesis in Huntington's disease

Andrés Miguez[1,2,3,4,5,13]*, Cinta Gomis[1,2,3,4,5¥], Cristina Vila[1,2,3,4,5¥], Marta Monguió-Tortajada[6], Sara Fernández-García[3,4,5,7], Georgina Bombau[1,2,3,4,5], Mireia Galofré[1,2,3,4,5], María García-Bravo[8], Phil Sanders[1,2,3,4,5], Helena Fernández-Medina[1,2,3,4,5], Blanca Poquet[1,2,3,4,5], Cristina Salado-Manzano[1,2,3,4,5], Santiago Roura[9,10], Jordi Alberch[2,3,4,5,7], José Carlos Segovia[8], Nicholas D. Allen[11], Francesc E. Borràs[6,12], Josep M. Canals[1,2,3,4,5]*

[1]Laboratory of Stem Cells and Regenerative Medicine, Department of Biomedical Sciences, Faculty of Medicine and Health Sciences, University of Barcelona, Barcelona, Spain.

[2]Creatio, Production and Validation Center of Advanced Therapies, Faculty of Medicine and Health Sciences, University of Barcelona, Barcelona, Spain.

[3]Institute of Neurosciences, University of Barcelona, Barcelona, Spain.

[4]August Pi i Sunyer Biomedical Research Institute (IDIBAPS), Barcelona, Spain.

[5]Networked Biomedical Research Centre for Neurodegenerative Disorders (CIBERNED), Spain.

[6]REMAR-IVECAT group, Germans Trias i Pujol Health Science Research Institute, Can Ruti Campus, Badalona, Spain.

[7]Laboratory of Pathophysiology of Neurodegenerative Diseases, Department of Biomedical Sciences, Faculty of Medicine and Health Sciences, University of Barcelona, Barcelona, Spain.

[8]Division of Hematopoietic Innovative Therapies, Centro de Investigaciones Energéticas, Medioambientales y Tecnológicas, Madrid, Spain.

[9]ICREC Research Program, Germans Trias i Pujol Health Science Research Institute, Can Ruti Campus, Badalona, Spain.

[10]Faculty of Medicine, University of Vic-Central University of Catalonia (UVic-UCC), Vic, Spain

[11]Brain Repair Group, School of Biosciences, Cardiff University, Cardiff, UK.

[12]Nephrology Department, Germans Trias i Pujol Universitary Hospital, Badalona, Spain.

[13]Present Address: Neurology-Neuroimmunology Department, Multiple Sclerosis Centre of Catalunya (Cemcat), Vall d'Hebron Research Institute (VHIR), Vall d'Hebron University Hospital, Barcelona, Spain.

[¥]Both authors contributed equally to the work.

***Corresponding authors:** andres.miguez@vhir.org (ORCID: 0000-0001-6014-3685)
jmcanals@ub.edu (ORCID: 0000-0001-6829-7670)

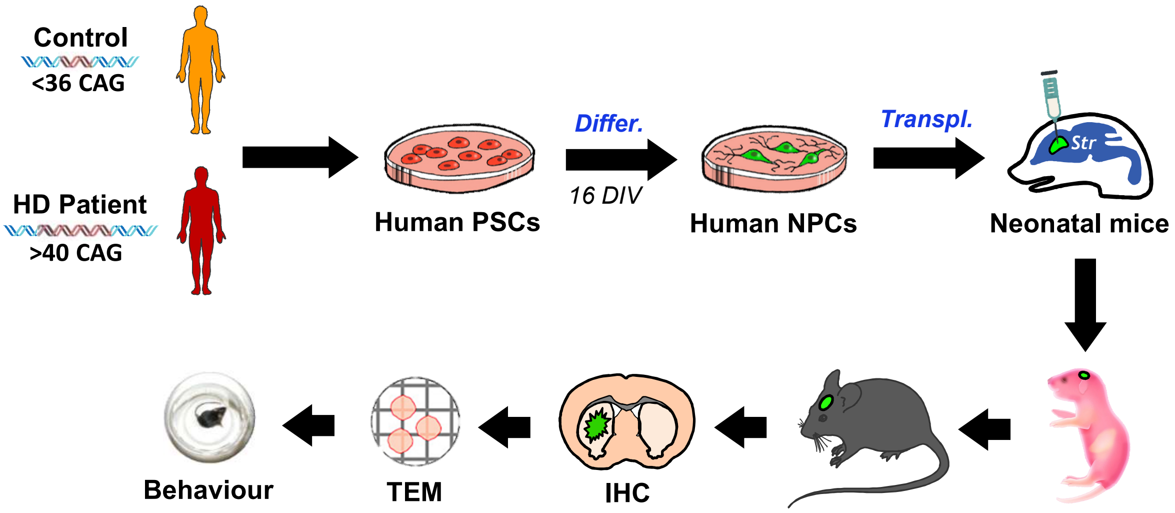

Supplementary Fig. 1 Workflow. Schematic cartoon depicting the workflow of the study. *PSCs*, pluripotent stem cells; *NPCs*, neural progenitor cells; *IHC*, Immunohistochemistry; *TEM*, Transmission electron microscopy

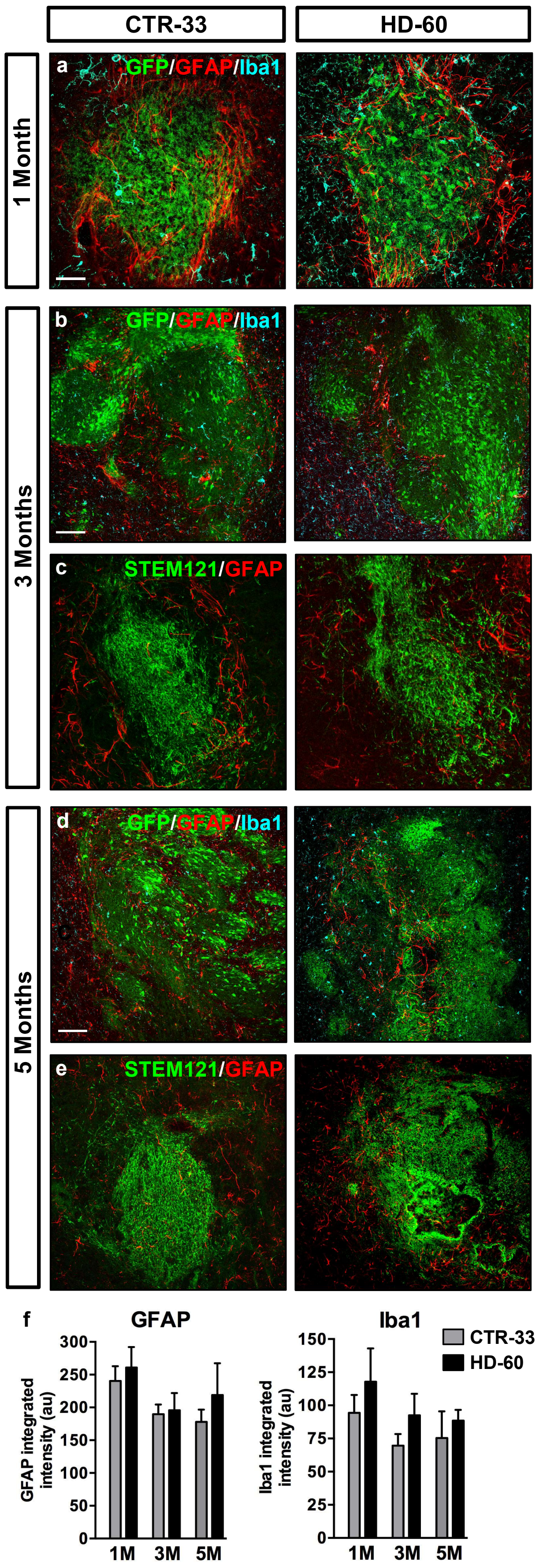

Supplementary Fig. 2 Immune response following transplantation of human neural progenitor cells. **a-e** Striatal coronal sections from CTR and HD chimeric mice at 1 (**a**), 3 (**b**, **c**) and 5 (**d**, **e**) months PST, immuno-labelled for GFP or STEM121 (green), GFAP (red) and Iba1 (cyan). **f** Histogram representing GFAP and Iba1 immunoreactivity around the graft. Scale bars: 50 µm in **a**; 100 µm in **b**, **d**. Data are expressed as mean ± SEM. **f** *n* = 5 mice; Unpaired t-test, two-tailed, non-significant

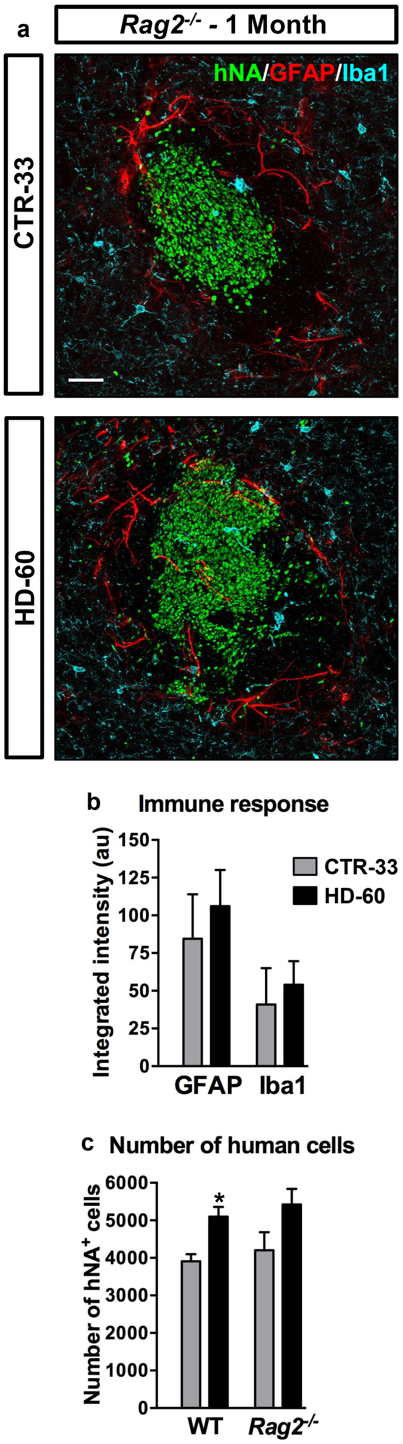

Supplementary Fig. 3 Transplantation of human neural progenitor cells in *Rag2*$^{-/-}$ immunodeficient mice. **a** Striatal coronal sections of *Rag2*$^{-/-}$ immunodeficient mice neonatally engrafted with CTR and HD cells, and immuno-labelled for hNA (green), GFAP (red) and Iba1 (cyan) at 1 month PST. **b** Histogram representing GFAP and Iba1 immunoreactivity around the graft at 1 month PST. **c** Histogram representing the number of surviving human cells in *Rag2*$^{-/-}$ mice compared to WT mice at 1 month PST. Scale bar: 100 µm. Data are expressed as mean ± SEM. **b** n = 3 *Rag2*$^{-/-}$ mice; Unpaired t-test, two-tailed, non-significant. **c** n = 5 WT mice, n = 3 *Rag2*$^{-/-}$ mice; Unpaired t-test, two-tailed, P = 0.0232 (WT), P = 0.1215 (*Rag2*$^{-/-}$)

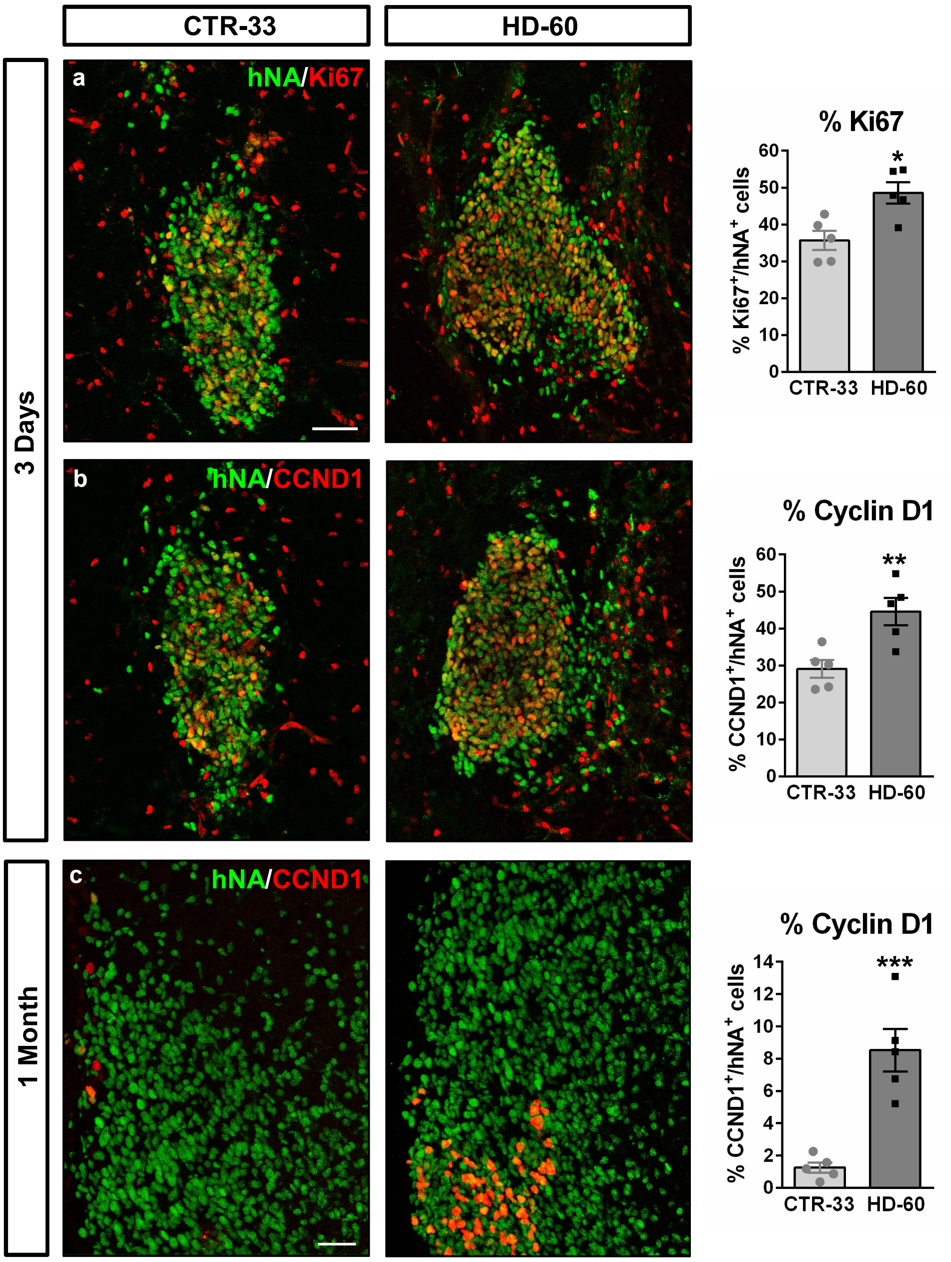

Supplementary Fig. 4 Neonatally engrafted HD patient-derived human neural progenitor cells show increased proliferation and cyclin D1 expression. **a-c** Striatal coronal sections from CTR and HD chimeric mice at 3 days (**a**, **b**) and 1 month PST (**c**), immuno-labelled for hNA (green) and Ki67 or cyclin D1 (CCND1) (red). Histograms show the percentage of Ki67^{+} proliferating cells (**a**) and CCND1^{+} cells (**b**, **c**). Scale bars: 50 µm. Data are expressed as mean ± SEM. **a** n = 5 mice, Unpaired t-test, two-tailed, P = 0.0106; **b** n = 5 mice, Unpaired t-test, two-tailed, P = 0.0077; **c** n = 5 mice, Unpaired t-test, two-tailed, P = 0.0007

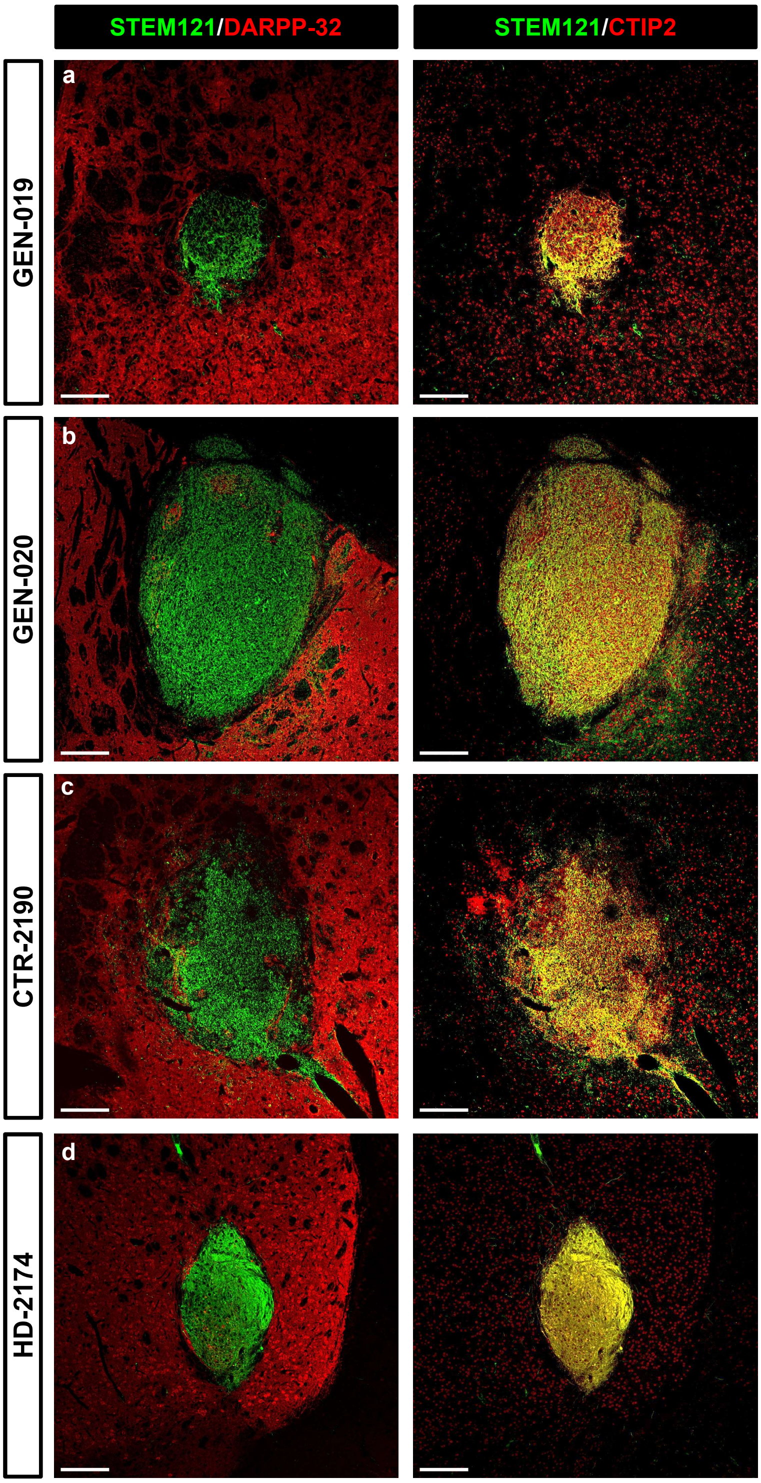

Supplementary Fig. 5 Neonatally engrafted GEN-019, GEN-020, CTR-2190 and HD-2174 human neural progenitor cells survive 3 months and differentiate into CTIP2^{+} striatal neurons. **a-d** Striatal coronal sections from CTR and HD chimeric brains at 3 months PST, immuno-labelled for STEM121 (green), and CTIP2 or DARPP-32 (red). The vast majority of transplanted cells show high CTIP2 expression. Scale bars: 200 µm. **a**, **c**, **d** *n* = 3 mice; **b** *n* = 4 mice

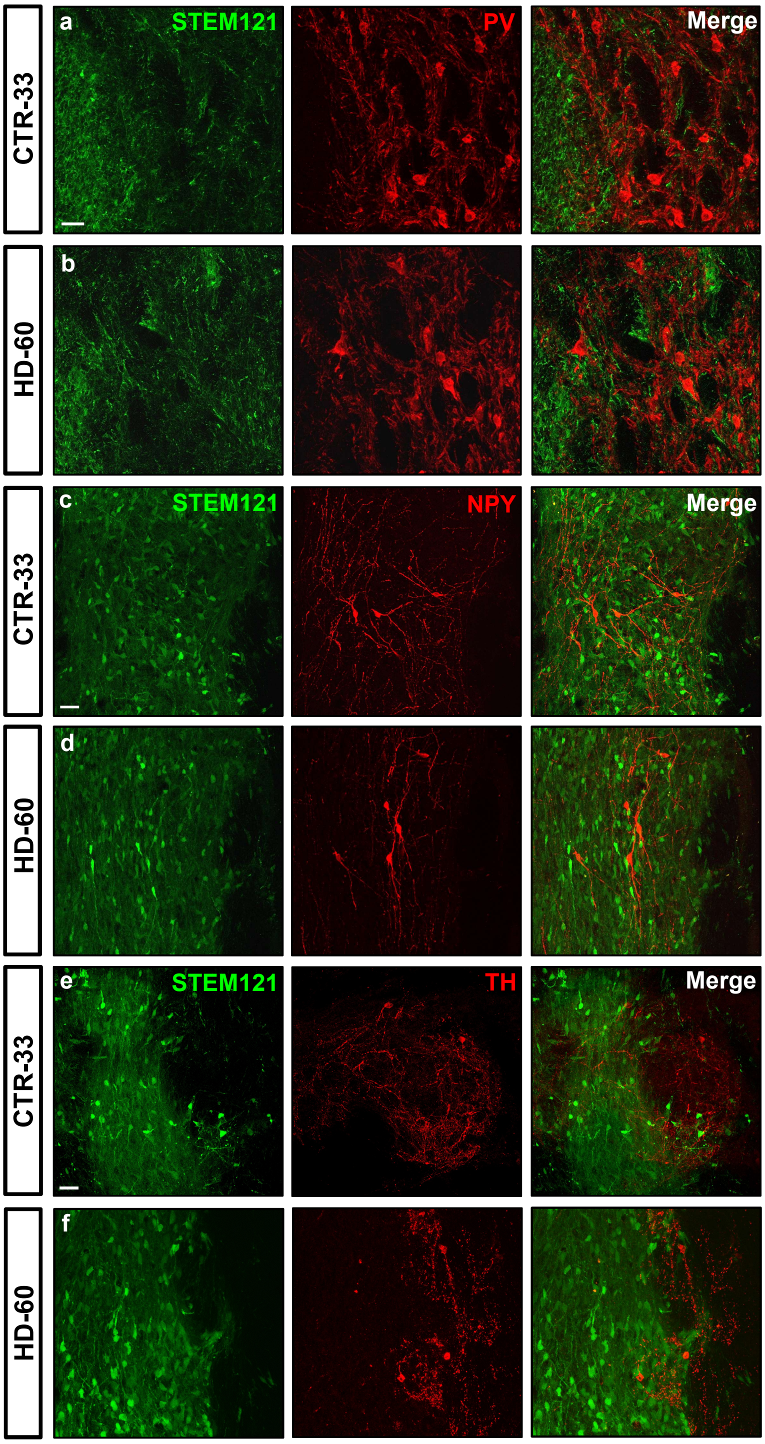

Supplementary Fig. 6 Absence of Parvalbumin, Neuropeptide Y and TH expression by engrafted human cells. **a-f** Striatal coronal sections of CTR and HD chimeric mice at 3 months PST immuno-labelled for STEM121 (green) and either Parvalbumin (PV) (**a**, **b**), Neuropeptide Y (NPY) (**c**, **d**) or Tyrosine hydroxylase (TH) (**e**, **f**) (red). Scale bars: 50 µm. $n = 4$ mice

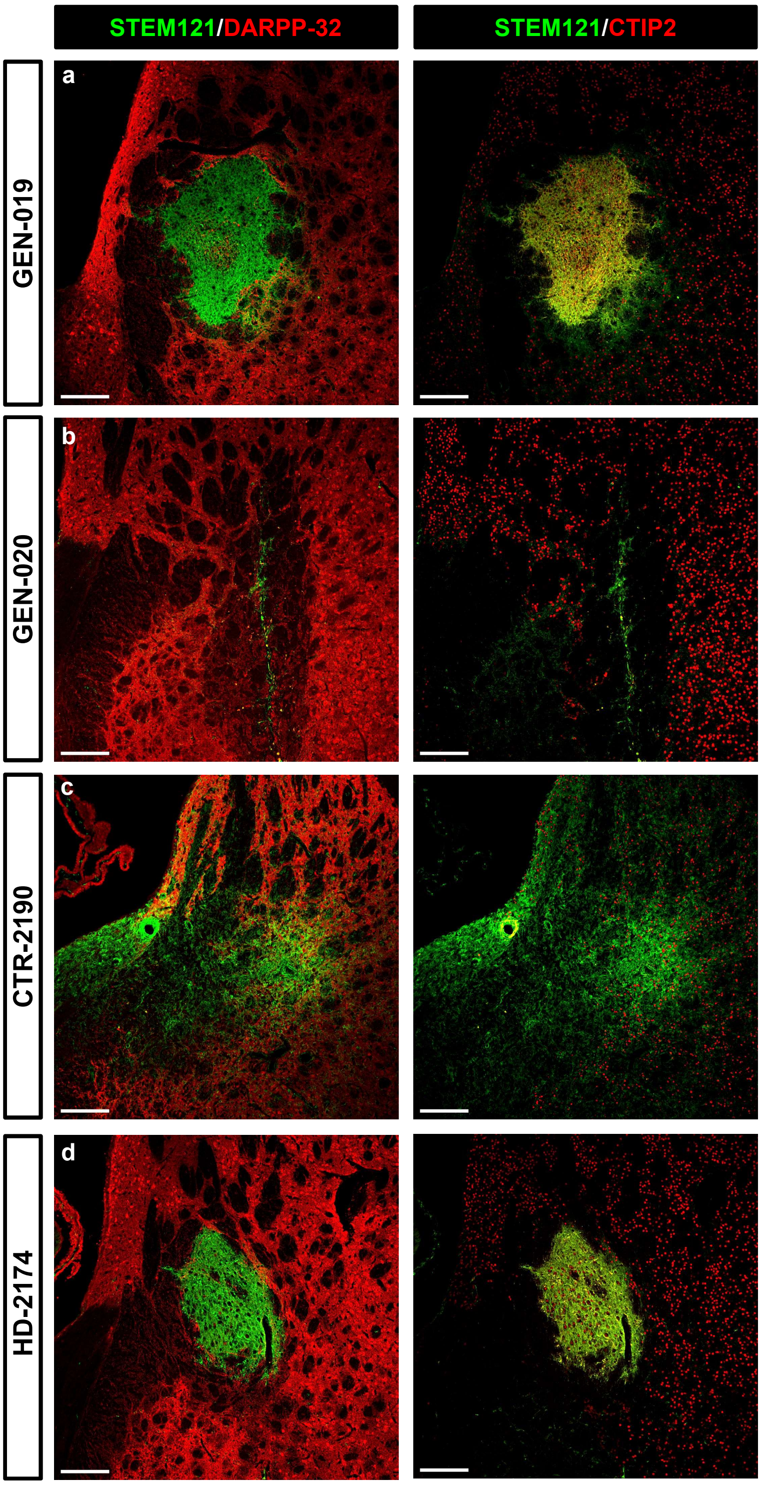

Supplementary Fig. 7 Neonatally engrafted human neural progenitor cells from GEN-019, GEN-020, CTR-2190 and HD-2174 cell lines survive up to 5 months post-transplantation. Striatal coronal sections from GEN-019 (**a**), CTR-2190 (**b**) and HD-2174 (**c**) chimeric brains at 5 months PST, immuno-labelled for STEM121 (green), DARPP-32 (red) and CTIP2 (red). Scale bars: 200 µm. *n* = 3 mice

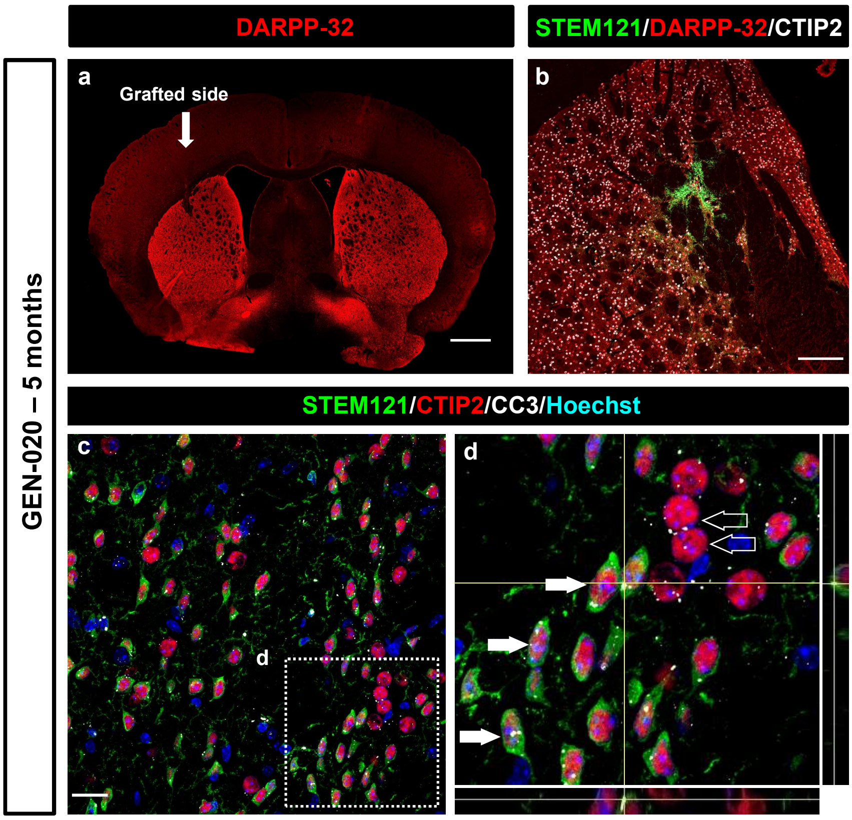

Supplementary Fig. 8 Neonatally engrafted GEN-020 human neural progenitor cells show striatal neurodegeneration at 5 months post-transplantation. **a, b** Striatal coronal sections from GEN-020 chimeric brains at 5 months PST, immuno-labelled for STEM121 (green), DARPP-32 (red) and CTIP2 (white). Note the increased ventricular volume on the grafted side (**a**) and the loss of striatal tissue (**b**). **c, d** Striatal coronal sections from HD chimeric brains at 5 months PST immuno-labelled for STEM121 (green), CTIP2 (red) and cleaved caspase-3 (white). Filled arrows point to apoptotic human striatal neurons and empty arrows point to apoptotic endogenous mouse cells. Scale bars: 1 mm in **a**, 20 µm in **b**, 200 µm in **c**. $n = 6$ mice

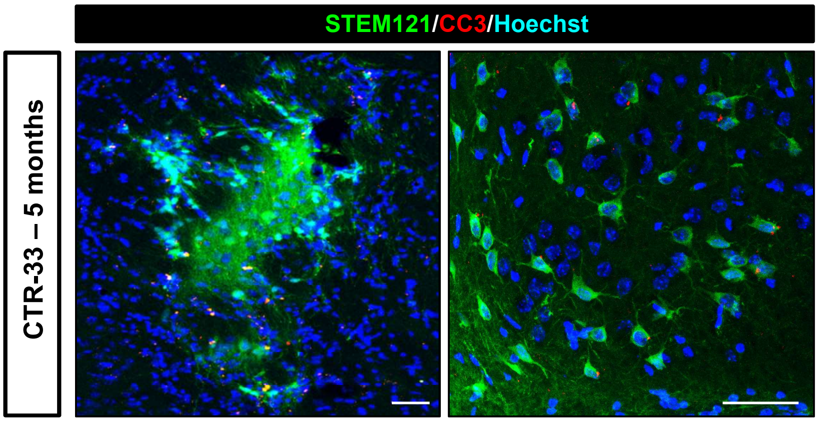

Supplementary Fig. 9 Neonatally engrafted CTR-33 human neural progenitor cells do not induce striatal neurodegeneration of mouse striatal cells at 5 months post-transplantation. Striatal coronal sections from CTR-33 chimeric brains at 5 months PST immuno-labelled for STEM121 (green), cleaved caspase-3 (red) and Hoescht. Scale bars: 50 µm. *n* = 5 mice

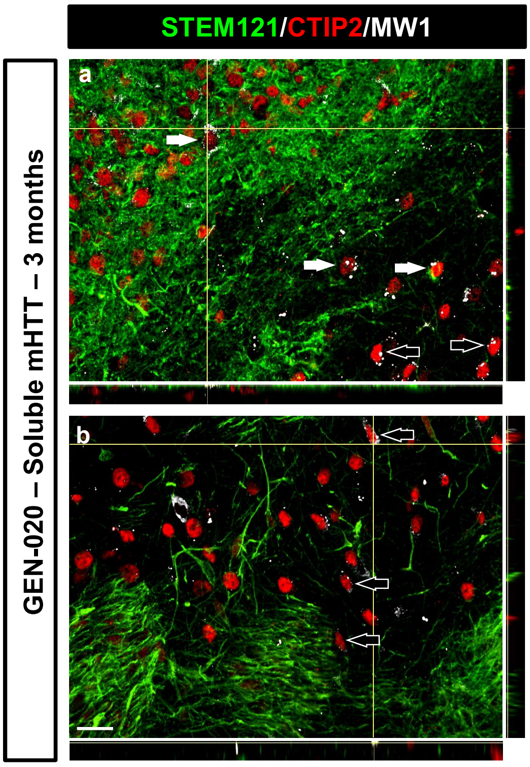

Supplementary Fig. 10 Neonatally engrafted GEN-020 human neural progenitor cells propagate soluble mHTT to mouse striatal neurons. Illustrative striatal coronal sections from a GEN-020 chimeric brain at 3 months PST immuno-labelled for STEM121 (green), CTIP2 (red) and MW1 (white). White arrows point to $MW1^+$ puncta inside $STEM121^+/CTIP2^+$ human striatal neurons, while black arrows point to $MW1^+$ puncta in $STEM121^-/CTIP2^+$ mouse striatal neurons. Scale bar: 20 µm. *n* = 4 mice

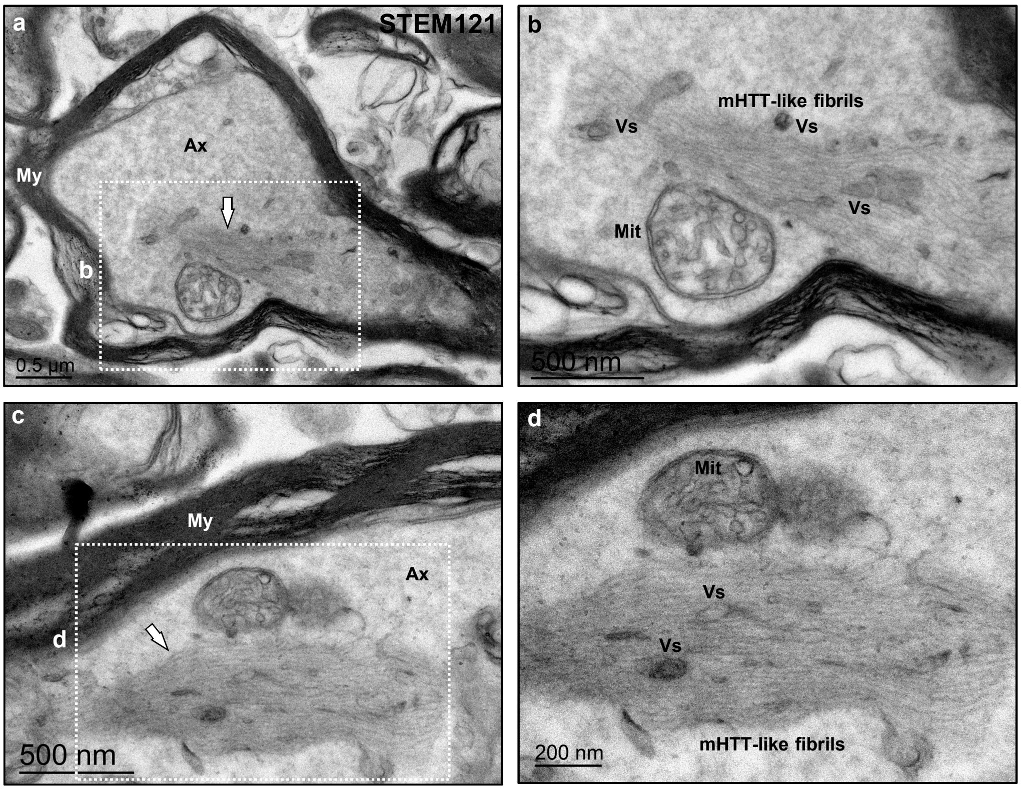

Supplementary Fig. 11 Presence of mHTT-like fibrils in axons from mouse host cells at 5 months PST. Ultra-thin sections of myelinated axons from a HD-60 chimeric brain at the striato-pallidal region at 5 months PST. STEM121^{-} axons (corresponding to mouse host cells) contain bundles of mHTT-like fibrils (arrows) in close proximity to mitochondria. Fibrils appear to be disrupting the outer double membrane of the mitochondria, which display disorganized cristae in their lumen. Note the presence of irregular vesicles trapped within the bundles (**b**, **d**), likely resulting from the disruption of organellar membranes following interaction with the fibrils, as previously described in Bäuerlein et al. [45]. *Ax*, axon; *My*, myelin; *Mit*, mitochondria; *Vs*, vesicles

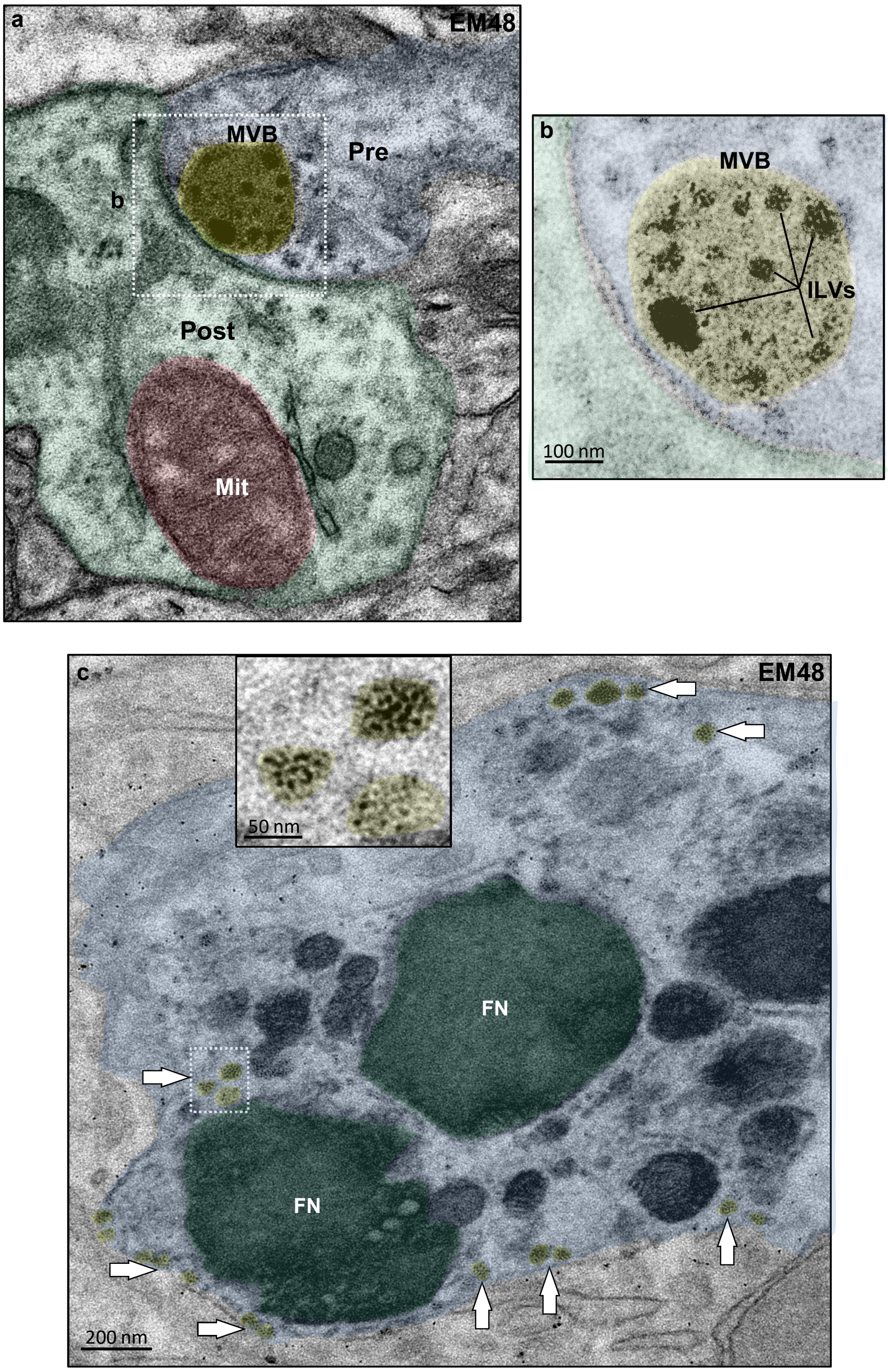

Supplementary Fig. 12 Presence of mHTT in multivesicular bodies at presynaptic terminals and within exosome-like vesicles inside dead cells of HD chimeric mice at 5 months PST. **a, b** Ultra-thin section from a HD-60 chimeric brain at the external globus pallidus (GPe) region, immunogold-labelled for EM48 and analysed by TEM. **c** Ultra-thin striatal section from a HD-60 chimeric brain immunogold-labelled for EM48 and analysed by TEM at 5 months PST. White arrows point to exosome-like vesicles containing EM48^{+} particles at the plasma membrane and cytoplasm of a dead cell with fragmented nucleus. *MVB*, multivesicular body; *ILVs*, Intraluminal vesicles; *Pre*, presynaptic terminal; *Post*, postsynaptic terminal; *Mit*, mitochondria; *FN*, fragmented nucleus

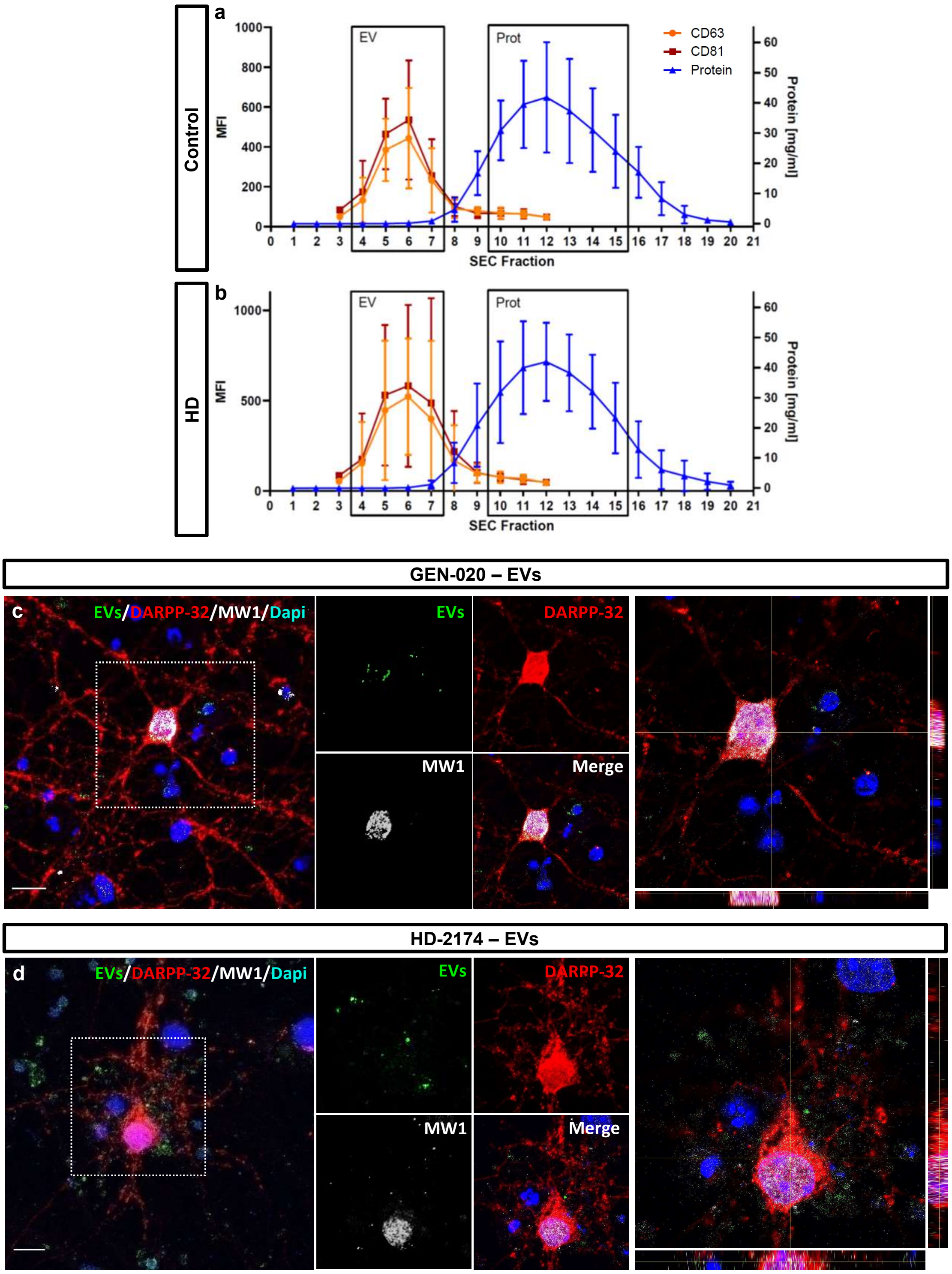

Supplementary Fig. 13 Isolation of extracellular vesicles secreted by human neural progenitor cells at 16 DIV and analysis of the soluble mHTT content of GEN-020 and HD-2174 EVs. **a, b** Size-exclusion chromatography (SEC) fractions of EVs and proteins isolated from the conditioned culture medium of CTR- (**a**) and HD-hNPCs (**b**) at 16 DIV and analysed by flow cytometry using CD63 and CD81 markers. **c, d** Co-culture of fluorescently labelled human EVs from GEN-020 and HD-2174 cell lines (green) with DARPP-32^{+} mouse primary striatal neurons (red), for 24h at a 2:1 ratio (EV-donor cells: EV-recipient cells). Note the presence of abundant MW1^{+} particles inside mouse cells. **a**, **b** $n = 3$ CTR and 3 HD cell lines; $n = 3$ independent in vitro differentiations. Scale bars: 10 μm

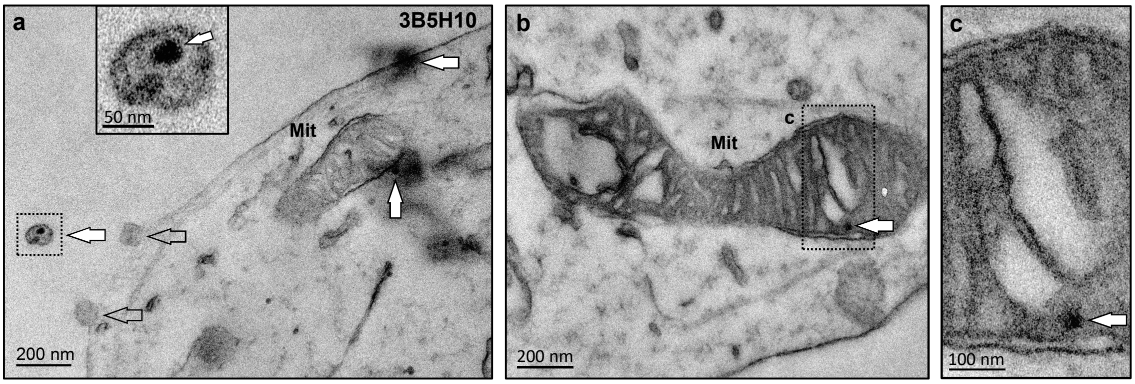

Supplementary Fig. 14 mHTT monomers within HD EVs can be propagated to mouse striatal neurons. Ultra-thin sections from co-cultures of HD-60 EVs with mouse striatal neurons immunogold-labelled for 3B5H10 and analyzed by TEM after 24h. Filled arrows point to 3B5H10^{+} mHTT monomers, both inside (inset in **a**) and outside EVs, while empty arrows point to EVs at the cell membrane devoided of mHTT monomers. Note the presence of 3B5H10^{+} small particles interacting with mitochondria inside mouse striatal neurons (**b**, **c**). *Mit*, mitochondria

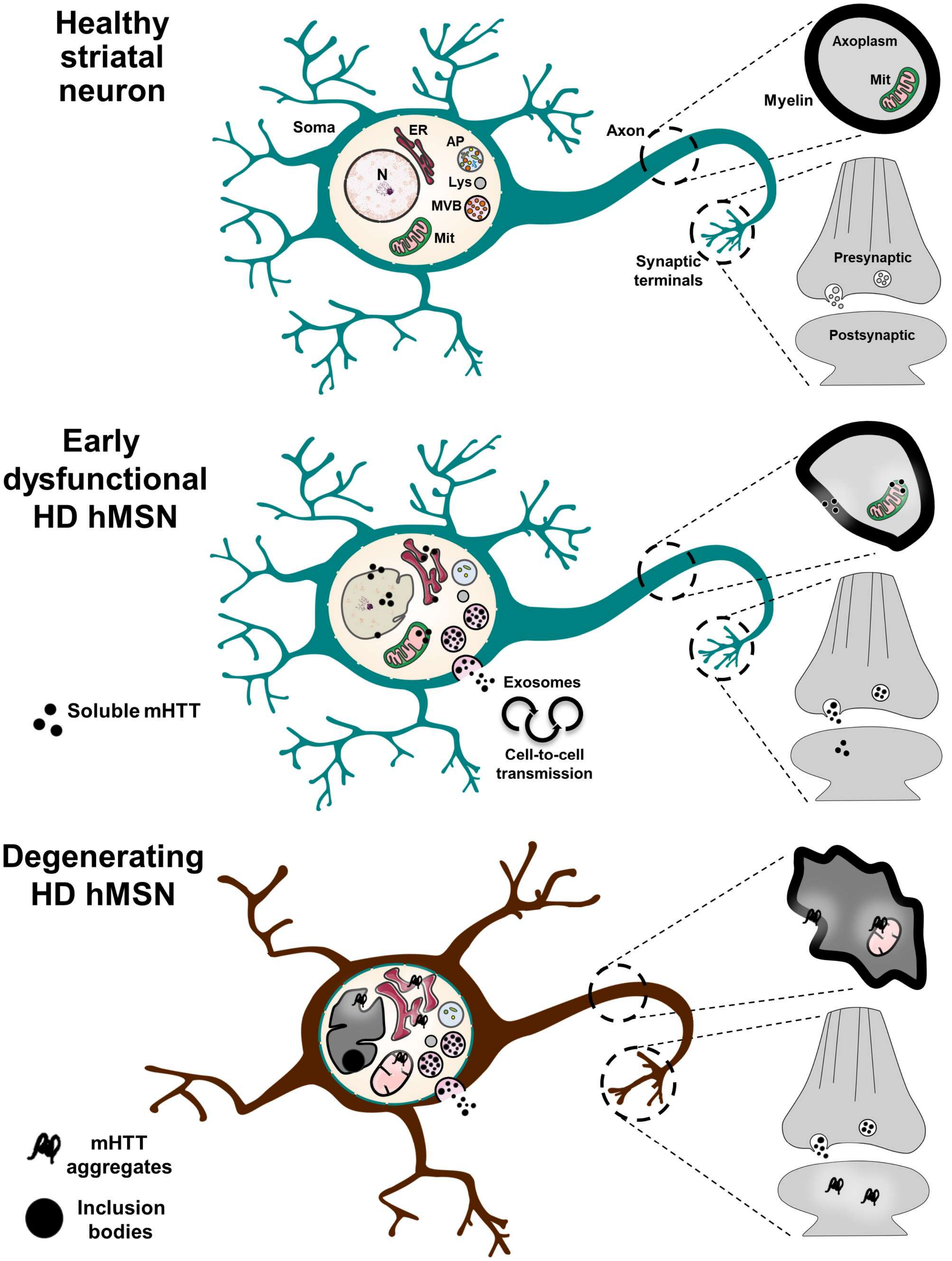

Supplementary Fig. 15 Summary of the mHTT-associated progressive alterations found in HD patient-derived human striatal neurons (see next page)

Supplementary Fig. 15 Summary of the mHTT-associated progressive alterations found in HD patient-derived human striatal neurons. During early phases of HD, the appearance of soluble mHTT in striatal neurons alters the structure of endoplasmic reticulum (ER), mitochondria (Mit) and nuclear (N) membrane in the cell soma. In addition, mHTT accumulates in multivesicular bodies (MVBs) before being degraded through the amphisome (AP)-lysosome (Lys) pathway or secreted within exosomes, the latter contributing to disease propagation. At the axonal level, mHTT mainly interacts with mitochondria, myelin sheath and synaptic terminals, inducing axonal degeneration. Degenerating striatal neurons develop small mHTT aggregates, but just a few form inclusion bodies. *hMSN*, human medium spiny neuron; *mHTT*, mutant huntingtin

a

Mice with detected grafts (%)		
**Cell line**	**3 months**	**5 months**
**CTR-33**	67%	75%
**HD-60**	73%	73%
**GEN-019**	33%	100%
**GEN-020**	50%	67%
**CTR-2190**	57%	50%
**HD-2174**	40%	67%

b

Graft Volume ($\times 10^6$ µm^3)	
**Cell lines**	**Mean ± SEM**
**Control**	95.4 ± 6.0
**HD**	1159.9 ± 594.2

Supplementary Table 1 Assessment of graft size and survival in cell transplantation experiments. Tables indicating the percentage of transplanted mice containing engrafted cells at 3 and 5 months PST (**a**) and the quantification of graft volume at 3 months PST for control cell lines CTR-33 and GEN-019 and HD cell lines HD-60, GEN-020 and HD-2174 (**b**)

Cell marker	Cell line	Time PST	Number of cells (Mean ± SEM)
NeuN	Control	1 Month	3168.7 ± 219.3
		3 Months	3908.4 ± 120.7
	HD	1 Month	4127.6 ± 181.9
		3 Months	5139.6 ± 117.5
Olig2	Control	1 Month	259.7 ± 103.0
		3 Months	197.2 ± 164.7
	HD	1 Month	349.1 ± 198.6
		3 Months	304.7 ± 173.7
CTIP2	Control	1 Month	3566.1 ± 135.8
		3 Months	3601.1 ± 342.4
		5 Months	2600.6 ± 184.1
	HD	1 Month	4624.9 ± 175.9
		3 Months	4015.2 ± 191.4
		5 Months	1299.2 ± 408.6
DARPP-32	Control	1 Month	290.9 ± 79.7
		3 Months	220.9 ± 56.6
		5 Months	165.3 ± 38.5
	HD	1 Month	1011.1 ± 203.5
		3 Months	558.3 ± 134.4
		5 Months	99.7 ± 36.0
Calretinin	Control	1 Month	174.8 ± 28.4
		3 Months	674.5 ± 122.1
		5 Months	1310.8 ± 160.4
	HD	1 Month	180.9 ± 63.5
		3 Months	1208.2 ± 165.5
		5 Months	1444.3 ± 301.3
hNA	Control	1 Month	3947.4 ± 192.7
		3 Months	4391.0 ± 560.9
		5 Months	4091.5 ± 411.8
	HD	1 Month	5038.5 ± 236.5
		3 Months	5622.2 ± 357.0
		5 Months	2977.1 ± 712.2

Supplementary Table 2. Number of human cells quantified in cell fate studies (refers to Fig. 1)

Antibody	Host	Dilution	Use	Source	Reference
**Calretinin**	Goat	1:1000	IHC	Millipore	ab1550
**Cleaved caspase-3**	Rabbit	1:500	IHC	Cell Signaling	9661S
**CD63**	Rabbit	1:2000	IHC	Abcam	ab134045
**CD63 (TEA3/18)**	Mouse	1:1000	Flow Cyt	Hybridoma	----
**CD81 (5A6)**	Mouse	1:1000	Flow Cyt	Hybridoma	----
**CTIP2**	Rat	1:200	IHC	Abcam	ab18465
**Cyclin D1**	Rabbit	1:200	IHC	Abcam	ab134175
**DARPP-32 (19A3)**	Rabbit	1:500	IHC, ICC	Cell Signaling	2306
**DARPP-32**	Mouse	1:1000	ICC	BD Biosciences	611520
**EM48**	Mouse	1:100<br>1:50*	IHC, ICC<br>TEM*	Millipore	MAB5374
**GFP-FITC**	Goat	1:500	IHC	Abcam	ab6662
**GFP**	Rabbit	1:500	TEM	Abcam	ab6556
**GFAP**	Mouse	1:500	IHC	Sigma	G3893
**GFAP**	Rabbit	1:1000	IHC	Dako	Z0334
**Human nuclei**	Mouse	1:200	IHC	Millipore	MAB1281
**Iba1**	Rabbit	1:500	IHC	Wako	019-19741
**Ki67**	Rabbit	1:250	IHC	Abcam	ab16667
**MAP2 (2a+2b)**	Mouse	1:200	IHC	Sigma	M1406
**MW1**	Mouse	1:1000<br>1:100*	IHC, ICC<br>TEM*	Hybridoma Bank	AB_528290
**NeuN**	Rabbit	1:500	IHC	Cell Signaling	24307
**Neuropeptide Y**	Rabbit	1:300	IHC	Abcam	ab30914
**Olig2**	Rabbit	1:200	IHC	Millipore	ab9610
**Parvalbumin**	Rabbit	1:1000	IHC	Swant	PV-27
**STEM121**	Mouse	1:200<br>1:100*	IHC<br>TEM*	Takara	Y40410
**TH**	Rabbit	1:200	IHC	Millipore	ab152
**3B5H10**	Mouse	1:100	TEM	Sigma	MABN821

Supplementary Table 3. List of primary antibodies employed during the experimental work

Supplementary Video 1 Amphetamine-induced circling behavior of HD chimeric mice at 5 months PST. Representative movie of a HD-60 chimeric mouse performing the amphetamine-induced rotation test at 5 months PST. Amphetamine was delivered intraperitoneally at a dose of 5 mg/kg. After 2 min of latency, the number of ipsilateral and contralateral turns were counted manually during 15 min. Note the increased number of ipsilateral turns towards the transplanted left-brain hemisphere, indicative of unilateral striatal degeneration

Supplementary Video 2 Amphetamine-induced circling behavior of control chimeric mice at 5 months PST. Representative movie of a CTR-33 chimeric mouse performing the amphetamine-induced rotation test at 5 months PST. Amphetamine was delivered intraperitoneally at a dose of 5 mg/kg. After 2 min of latency, the number of ipsilateral and contralateral turns were counted manually during 15 min. Note that the animal shows a similar number of ipsilateral and contralateral turns
